# Segregation and Crosstalk of D1 Receptor-Mediated Activation of ERK in Striatal Medium Spiny Neurons upon Acute Administration of Psychostimulants

**DOI:** 10.1371/journal.pcbi.1003445

**Published:** 2014-01-30

**Authors:** Omar Gutierrez-Arenas, Olivia Eriksson, Jeanette Hellgren Kotaleski

**Affiliations:** 1 School of Computer Science and Communication, KTH Royal Institute of Technology, Stockholm, Sweden; 2 Department of Numerical Analysis and Computer Science, Stockholm University, Stockholm, Sweden; 3 Department of Neuroscience, Karolinska Institute, Stockholm, Sweden; Research Center Jülich, Germany

## Abstract

The convergence of corticostriatal glutamate and dopamine from the midbrain in the striatal medium spiny neurons (MSN) triggers synaptic plasticity that underlies reinforcement learning and pathological conditions such as psychostimulant addiction. The increase in striatal dopamine produced by the acute administration of psychostimulants has been found to activate not only effectors of the AC5/cAMP/PKA signaling cascade such as GluR1, but also effectors of the NMDAR/Ca^2+^/RAS cascade such as ERK. The dopamine-triggered effects on both these cascades are mediated by D1R coupled to Golf but while the phosphorylation of GluR1 is affected by reductions in the available amount of Golf but not of D1R, the activation of ERK follows the opposite pattern. This segregation is puzzling considering that D1R-induced Golf activation monotonically increases with DA and that there is crosstalk from the AC5/cAMP/PKA cascade to the NMDAR/Ca^2+^/RAS cascade via a STEP (a tyrosine phosphatase). In this work, we developed a signaling model which accounts for this segregation based on the assumption that a common pool of D1R and Golf is distributed in two D1R/Golf signaling compartments. This model integrates a relatively large amount of experimental data for neurons in vivo and in vitro. We used it to explore the crosstalk topologies under which the sensitivities of the AC5/cAMP/PKA signaling cascade to reductions in D1R or Golf are transferred or not to the activation of ERK. We found that the sequestration of STEP by its substrate ERK together with the insensitivity of STEP activity on targets upstream of ERK (i.e. Fyn and NR2B) to PKA phosphorylation are able to explain the experimentally observed segregation. This model provides a quantitative framework for simulation based experiments to study signaling required for long term potentiation in MSNs.

## Author Summary

The molecular mechanisms by which a dog learns to associate a bell ring with incoming food and by which addiction to cocaine is developed have many things in common. In both cases, glutamate-mediated inputs from the cortex converge on striatal neurons with the neuromodulator dopamine whose level increases upon food delivery or cocaine administration. This convergence triggers intracellular reaction cascades that end up modifying the basal state of these neurons into what is currently considered the cellular correlate of learning and its overblown variant: addiction. Understanding these signaling cascades is required to design interventions for the enhancement of cognitive capacities and the prevention/cure of addiction. With this in mind, we developed a model of the dynamics of these reaction cascades upon the administration of cocaine. We did this by gathering the cascades' molecular components and interactions discovered by experimentalist so far and then setting the reaction parameters so that the model reproduces the timing of the molecular changes that lead to the discovery of those components and interactions. With this integrationist endeavor we disclosed some inconsistencies and provided a rationale for experimental observations that were unexplained. Our predictions are readily challengeable with future experiments.

## Introduction

The interplay between dopamine and glutamate in the striatum is considered to mediate the role of this basal ganglia structure in reinforcement learning and action selection [1]. Glutamatergic projections arising from the cortex carry environmental information (a context, a cue or an action) into the striatum where dopamine is released by afferents from the midbrain. This dopamine signal occurs in response to salient events such as unexpected rewards [2], [3]. The glutamatergic and dopaminergic inputs converge on striatal medium spiny neurons (MSN) where they trigger neuronal plasticity mechanisms that allow the animal to associate the salient event and the environment that preceded it. This system is hijacked by psychostimulants like cocaine and amphetamines, which acting directly on the dopaminergic terminals generate an increase in the striatal dopamine levels [4] so that the context paired with the administration of the drug is actively sought by the animal even after just a single trial [5]. This behavior is considered to be a correlate of addiction in humans.

The elucidation of the receptor-induced signaling cascades taking place in the neurons of the circuitry integrating dopaminergic and glutamatergic inputs is considered a natural requirement for the design of effective pharmacological treatments for preventing/curing addiction. In this regard, it has been found that acute psychostimulant administration (**APA**) to naïve animals produces conspicuous molecular phenotypes in dopamine type 1 receptor (D1R) expressing MSNs (D1R+MSN) which constitute half of striatal MSNs [6]–[8] (throughout this work we use a broad definition of phenotype [9]–[11] that comprises not only macroscopic observables in mutant and wild type living animals upon some treatment like APA, but also traits in samples from these animals like immunoblot bands from striatal slices, microscopy images and membrane currents).The co-stimulation with dopamine and glutamate has been found to be required for the activation of the mitogen activated protein kinase ERK2 (hereafter just ERK) [12]. Classically, this convergence is also required for the mobilization of GluR1-containing AMPAR (2-Amino-3-(3-hydroxy-5-Methyl-isoxazol-4-yl)Propanoic Acid Receptor) to the postsynaptic density through the dual phosphorylation of GluR1 by PKA and PKC/CaMKII [13], [14] but just the PKA site has been found to be modified in the psychostimulant paradigms [13], [15]. The phosphorylation of ERK and GluR1 are part of plasticity mechanisms which result in measurable behaviors [16], [17].

**Figure 1:**
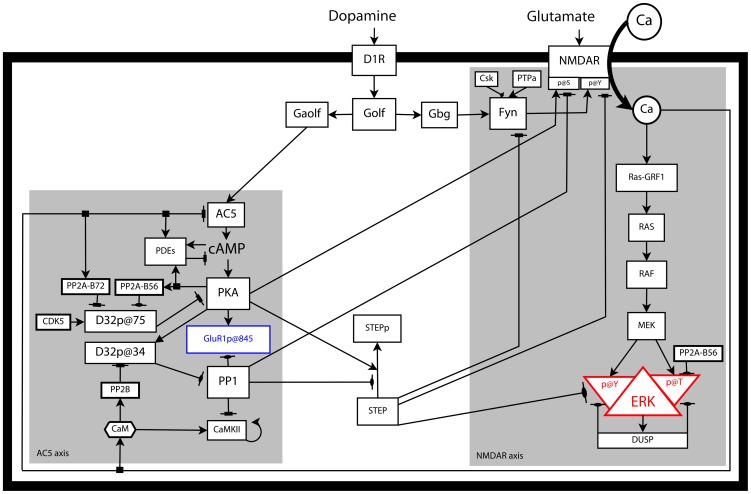
Graphical representation of the standard network modeled in this work. GluR1 (blue) and ERK (red) are the two effectors commonly used to monitor activity in the AC5 and NMDAR signaling cascades (axes), respectively. These signaling axes are depicted over a grey background. Detailed sub-networks are shown in Fig. S1 (Supporting Information).

The effects of APA in D1R+MSNs are mediated through two signaling cascades, the AC5 (Adenylyl Cyclase type 5)/cAMP/PKA axis (AC5 axis) and the NMDAR (N-Methyl-D-Aspartate Receptor)/Ca^2+^/RAS (NMDAR axis). These are both activated by dopamine signaling via D1R coupled to Golf. The AC5 axis is activated by the Golf α subunit [18] and the NMDAR axis is sensitized to glutamatergic input by the Golf βγ dimer [19] (Fig. 1). In order to study this system, mice with single copies of the genes encoding D1R (*Drd1a*^+/−^) and Gαolf (*GnaI^+/−^*) have been used. In both cases these animals have shown clear signs of haploinsufficiency (reduced amount of the gene product), with 20% and 40% of the wild type (WT) striatal levels of D1R and Gαolf (and thereafter the Golf heterotrimer)[20], respectively. APA experiments with these mutant mice have shown that the phosphorylation of GluR1 and ERK are associated to different sets of behaviors and that both the phosphorylation and the associated behaviors are *segregated* as explained in more detail below.

The AC5 axis comprises the classical generation of cAMP by adenylyl cyclase (AC5) with the consequent activation of PKA and inhibition of its counteracting PP1 via DARPP32 (D32) (Fig. 1). The PKA phosphorylation of GluR1 results in the exocytosis of GluR1-containing AMPA to the membrane and increases in single channel currents [14]. While no changes in the membrane levels of GluR1 has been found in the APA paradigm [15], the PKA phosphorylation of GluR1 *correlates* with the locomotor activation observed upon APA. The phosphorylation of GluR1 upon APA and the behavior associated to it are affected in *GnaI^+/−^* mice but neither in *Drd1a*^+/−^ mice [11], [15] nor in mice treated with a MEK inhibitor that prevents the activation of ERK [21] (APA-phenotypes in Table 1).

**Table 1:** Quantitative phenotypes used to constrain and challenge the model.

	Name	Treatment	Marker	Value	Ref.
**non-APA phenotypes**	basal	-	cAMP	60 nM	[23], [121]
			STEPact	80%	[12]
			D32p34	0.2–0.5 uM	[122]
			D32p75	13 uM	[123]
	DAsliceD32	Striatal slices+DA≥10 uM Sampled at 5′.	D32p34	12 X basal	[6], [124]
			D32p75	0.5 X basal	
	NMDAsliceD32	Striatal slices+NMDA 100 uM Sampled at 10′.	D32p34	0.5 X basal	[40], [125]
			D32p75	0.5 X basal	
	activateRAS	1″ Ca^2+^ spikes in cult. hipp. cells.	RAS•RAF complex	Hill. h = 4.1 K = 0.8 uM	[49]
	sensitizedNMDAR	NMDA ± SKF38393 3 uM in cult. MSNs. Sampled at 10′	ERKpp	Qualitative	[19]
	trafficNMDAR	PFC slices+DA. t series.	NMDAR currents	Monoexpon.k = 0.15 min^−1^	[61]
		Striatal slices+EtOH. t series.			[60]
**APA-phenotypes**	APAib	APA to WT mice. IB. t series	ERKpp	Fig. 4D	[12]
			GluR1p	Fig. 4F	[12], [13]
	haploD1R	APA to *Drd1a*^+/−^ mice. Sampled at 15′.	ERKpp	0.5 X WT	[19]
			GluR1p	1 X WT	
	haploGolf	APA to *GnaI^+/−^* mice. Sampled at 15′.	ERKpp	0.9 X WT	[20]
			GluR1p	0.6 X WT	
	D32KO	APA to D32KO mice. Sampled at 15′.	ERKpp	0.4 X WT	[12]
			GluR1p	0.35 X WT	[13]

STEPact, non-phosphorylated STEP over total STEP; D32p34, DARPP32 phosphorylated in threonine 34; D32p75, DARPP32 phosphorylated in threonine 75; PFC, prefrontal cortex; APA, acute psychostimulant administration; WT, wild type; IB, immunoblot; ERKpp, active ERK; GluR1p, AMPAR subunit GluR1 phosphorylated in the PKA site; t. series, time series; Monoexp, monoexponential. The names of the phenotypic variables are built from the name and marker columns except for parameterized time series or dose responses where it is used the parameter name instead of the marker.

In the NMDAR axis dopamine enhances NMDAR-mediated Ca^2+^ entry which results in the downstream activation of ERK through the RAS/RAF/MEK/ERK cascade. The activation of ERK, which triggers the expression of several genes, has been found to be essential for the development of conditioned place preference and locomotor sensitization which are considered behavioral signatures of addiction to psychostimulants [5], [21]. Furthermore, the activation of ERK has been found to mediate APA-induced LTP in D1R+MSN [22]. The activation of ERK upon APA and the behaviors associated to it are affected in *Drd1a*^+/−^ mice but not in *GnaI^+/−^* mice (APA-phenotypes in Table 1) [11], [14].

The AC5 and the NMDAR signaling axes have several crosstalking edges (Fig. 1). The Ca^2+^ entering through NMDAR activates serine/threonine phosphatases regulating the phosphorylation of D32 and inhibits AC5 activity [23]. On the other hand, the broadcast from the AC5 axis to the NMDAR axis is mediated by the tyrosine phosphatase STEP (Striatal Enriched tyrosine Phosphatase) which exists in two forms and is claimed to be inactivated by PKA-mediated phosphorylation which is counteracted by PP1 [24]–[26]. The phosphatase activity of STEP counteract the activation of Fyn, NMDAR and ERK in the NMDAR axis [26]. The significantly lower activation of not only GluR1 but also ERK upon APA in D32KO mice, where PP1 is not inhibited and the STEP phosphorylation by PKA is affected, has been interpreted as an evidence of the PKA-sensitive crosstalk mediated by STEP [12].

The segregation of the effects in *GnaI^+/−^* and *Drd1a*^+/−^ mice into the AC5 and the NMDAR axes is puzzling for at least two reasons. One is that as the D1R-catalyzed Golf activation monotonically increases with dopamine levels, thus the effectors that are changed in one mutant should be the same or a subset of the effectors that are changed in the other mutant. The other is that the crosstalk between the axes is expected to have a homogenizing effect by transferring the sensitivities from one axis to the other [27]. How can the segregation arise in the first place? Then, how is it maintained in the face of crosstalk? In this work, we have developed a quantitative signaling model which explains this data pattern. In this model the segregation arises from the distribution of a common pool of D1R and Golf in two D1R/Golf signaling compartments according to the affinity and capacity of compartment anchors. Each D1R/Golf compartment is dedicated to only one signaling axis. The segregation is kept in the face of crosstalk if the latter involves just a single pool of STEP whose activity is affected by PKA phosphorylation in a substrate dependent manner. These assumptions are backed by an increasing body of experimental support [24], [25], [28]–[32].

## Methods

### Network building

The model developed in this work integrates a substantial portion of the intracellular signaling triggered in MSNs by dopamine acting on D1R and glutamate on NMDAR. Two main signaling axes with several crosstalking edges are considered both of which are modulated upstream by dopamine via D1R coupled to Golf. One is the classical generation of cAMP by AC5 with the consequent activation of PKA and inhibition of its counteracting PP1 via D32. The other is the enhancement of NMDAR-mediated Ca^2+^ entry which results in the downstream activation of ERK. A detailed description follows (Fig. 1). This signaling cascade is triggered in dendritic spines and most of the processes occur there. The entire reaction network is modeled in a single well-stirred volume using ordinary differential equations (ODEs) that describe species concentration changes over time. However, the model includes the representation (through the indexing of species) of a few reaction compartments which stand for segregated reaction sites located in the spine such as the post-synaptic density (PSD), an internal NMDAR reservoir and two D1R/Golf signaling compartments. A few others processes which are part of the DUSP (dual specificity phosphatases)-mediated negative feedback loop inactivating ERK occur in other locations like the dendritic shaft, the soma and the nucleus and but these locations were not considered explicitly.

#### AC5/cAMP/PKA axis

Around 80% of this subnetwork was reconstructed and modeled in a previous work from our group [23]. Parameters values were kept the same whenever possible. The changes introduced are based on the publication of new experimental evidences and a reanalysis of older publications and procedures. The interaction between Golf and AC5 is now modeled stronger based on independent reports showing it to be in the low nanomolar range [33], [34]. Phosphodiesterases were also updated (Fig. S1A). Besides PDE1 and PDE4a considered in the previous model we introduce PDE10a, which is activated by cAMP [35]. PDE4a and PDE10a have been described to play a role in the attenuation of dopamine-induced cAMP generation in D1R+MSNs [36].

The ultrasensitive activation of PP2A by Ca^2+^ and the existence of a single PP2A pool considered in the previous model were replaced by a single site Ca^2+^ activation of a PP2A carrying the regulatory B72 subunit [37] which together with the PKA-sensitive B56-PP2A [38] constitute the two pools of PP2A included in this model and others [39]. The Ca^+2^ activation of B72-PP2A is substrate specific [37] and while Ca^2+^ increases the activity against D32p75, it does not seem to affect it toward D32p34 [40].

#### NMDAR/Ca^2+^/RAS axis

This axis constitutes a new addition to the model. Electrical stimulation of cortical neurons projecting to the striatum has been found to trigger in MSNs the upregulation of products like *fos* via an NMDAR-dependent process [41]. The upregulation of *fos* with this stimulation protocol is mediated by the activation of ERK [42]. Similarly, the activation of ERK in D1R+MSN by psychostimulants has been found to be dependent on both NMDAR and D1R stimulation [12], [21]. The NMDAR requirement for ERK activation in these neurons is mediated by Ca^2+^ entry through this ligand-gated channel [19] with the enrollment of RAS-GRF1 [43], a GEF (Guanine Nucleotide Exchange Factor) expressed in MSNs [44] and known to form a functional complex with the NR2B subunit of NMDAR [44], [45]. Reduction or elimination of the RAS-GRF1 function markedly affects the activation of ERK upon APA [43], [44]. The activation of RAS by Ca^2+^ bound RAS-GRF1 was modeled according to the paradigm where GEF speeds up nucleotide release by around 10000 times and stabilizes the nucleotide free form of the GTPase [46], [47]. This complex has equal preference for GTP and GDP, therefore the binding of the 10 times more abundant GTP [48] is predominant (Fig. S1B). In pyramidal neurons from the hippocampus, where ERK activation by NMDAR Ca^2+^ is also mediated by RAS-GRF1, the activation of RAS by Ca^2+^ pulses in dendritic spines is ultrasensitive (Hill ∼4) [49] and we incorporated this behavior in the activation of RAS-GRF1 which requires Ca^2+^-Calmodulin binding [50]. RAS-GTP activates RAF, the upper tier of the RAF/MEK/ERK cascade which we took from DOQCS (Database of Quantitative Cellular Signaling) [51], [52]. The tonic dephosphorylation of ERK was modified from being catalyzed in a distributive scheme by a dual specificity phosphatase to one where the tyrosine phosphatase STEP [53] and the serine/threonine phosphatase B56-PP2A [54] act in concert [55]. Phosphorylation of STEP by PKA is suggested to reduce its activity on phosphotyrosine ERK (Y187) but there is no indication that phosphorylation by PKA affects the activity of B56-PP2A on phosphothreonine ERK (T185).

The duration of ERK activation by psychostimulants was modeled to be limited by the action of inducible DUSPs. Many of these enzymes form part of a negative feedback loop triggered by the activation of MAPKs [56]. DUSP1, which has ERK as one of its substrates, albeit not the most efficient, has been found to be upregulated in the striatum in an ERK dependent way by electrical stimulation of corticostriatal projections [42]. DUSP1 and other DUSPs are also upregulated upon acute administration of methamphetamine [57] and its derivative MDMA [58] in a D1R dependent way.

#### DA/D1R enhancement of NMDAR Ca^2+^ currents

Dopamine acting through D1R has long been known to stimulate NMDAR currents [59] in a process mediated by serine/threonine and tyrosine phosphorylation of different subunits of NMDAR. In this model three different mechanism of DA/D1R enhancement of NMDAR Ca^2+^ currents were considered. In all cases NMDAR was represented by a single species with different phosphorylation states and two different localizations, synaptic and intracellular. We have assumed that the different phosphorylations are independent of each other and also independent of the location of the receptor. These modifications, which are triggered by DA/D1R/Golf signaling, result in increased NMDAR Ca^2+^ currents according to a factor that scales up the amplitude of basal Ca^2+^ transients (see below). While the signaling networks underlying each of these three mechanisms are operating concomitantly, the contribution of each of them to the dynamics of downstream effectors is probed independently. The absolute value of the scaling factor (Equations 1, 2 and 3 below) for each of these three mechanisms is arbitrary, thus the scaling factors were normalized to 1 in basal conditions (t = 0). Furthermore, the maximum amplitude of the scaling factors achieved by dopamine stimulation in this work is set to 2.5 in the three cases. The normalization and the common maximum amplitude allow comparison among the three different mechanisms of NMDAR enhancement in terms of time course. The selection of 2.5 as the maximum amplitude carries a dose of uncertainty. The scaling produced by the Fyn-mediated traffic-based mechanism has been found to be around 1.5 for EPSPs amplitude [60], [61]. However, electrophysiological measurements can underestimate increases in Ca^2+^ influx by enhancement of NMDAR function [62]. Higher values of maximum scaling factor amplitude are easily accommodated by the model, with no changes in the conclusion of this work.

The computation of each scaling factor is mechanism-dependent and it is performed as follows,

- PKA-mediated enhancement of single channel activity (sSCh): This is a fast mechanism triggered by DA acting through the AC5 axis. PKA phosphorylation of NMDAR has been found to increase Ca^2+^ currents through NMDAR [62]. The phosphorylation by PKA, possibly of the NR1 subunit, increases NMDAR whole cell currents in acute preparation of dissociated MSNs and this is reversed by PP1 [63]. The serine phosphorylation of the NR1 subunit induced in MSNs by D1R agonists in vivo is significantly reduced in DARPP32 knock-out mice [64] and similar effects were measured in striatal slices treated with dopamine [65]. Thus, this effect was modeled as PKA/PP1 acting on NMDAR. The scaling factor (scaleCa_s_) is computed as a function of the ratio of PKA-phosphorylated NMDAR (NMDARmp@S) over total membrane NMDAR (NMDAR_m_),
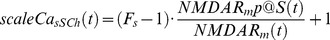
(1)where Fs is the fold increase in conductance upon PKA phosphorylation but in practice it was set to have an effect whose amplitude is of similar size than the other two mechanisms (see above) for comparison of the time courses.
- Fyn-mediated increase of NR2B-containing synaptic NMDAR (yTrf): This is a relatively slower traffic-based mechanism were DA acting through D1R promotes an increase in NR2B-dependent synaptic NMDAR currents. Both NR2A and NR2B are the most abundant NR2-type NMDAR subunits in the striatum [66] but the NR2B subunit convenes a higher total conductance [67] and Ca^2+^ permeability than NR2A [68]. This effect is mediated by the phosphorylation of the Y1472 residue in NR2B by the Src-like non-receptor protein tyrosine kinase Fyn [69]. NR2B-p@Y1472 is dephosphorylated by STEP [70]. The phosphorylation of Y1472 in NR2B prevents the binding of the AP2 clathrin adaptor and the endocytosis by this mechanism and thus indirectly results in an increase of the NR2B containing NMDAR in the cell membrane [71], [72] and its inclusion in the synapse [73]. Fyn mediated tyrosine phosphorylation also enhances the insertion of NR2B subunits in the membrane upon D1R stimulation in striatal MSNs [64], [74]. We modeled the effects of Y1472 phosphorylation in the synaptic density of NR2B-containing NMDAR with a faster exocytosis rate for the phosphorylated NMDAR than for the non-phosphorylated one, together with an endocytosis that operates just on the non-phosphorylated membrane bound NMDAR. Fyn and STEP act on both the intracellular and the synaptic NMDAR. The activity of Fyn is regulated by the phosphorylation state of two of its tyrosine residues, Y420 and Y527 (Fig. S1C). Y420 is phosphorylated through a second order autophosphorylation [75] and dephosphorylated by STEP [76]. Y527 is phosphorylated by Csk and dephosphorylated by PTPa. Of the four possible combinations of phosphorylation in these two residues, just Fyn singly phosphorylated in Y527 is inhibited [75]. The three other combinations have similar activities [75], [77]. Autophosphorylation of Y420 is prevented if Y527 is phosphorylated but the reciprocal does not hold as Csk can still phosphorylate Fyn-p@420 [75]. While the activities of Csk and PTPa were kept at a constant basal level in the model [78], the activities of Fyn-p@Y527 and STEP were regulated by dopaminergic and glutamatergic signaling. Dopamine acting on D1R was found to activate Fyn (increased Y420 phosphorylation) via G_βγ_ subunits but the topology of this link has not been found yet [19]. We assumed that a direct interaction between G_βγ_ and Fyn-p@527 allowed the second order autophosphorylation which generates an active bi-phosphorylated Fyn. A similar point of activation of Src-like kinases by GPCR signaling, promoting autophosphorylation of @Y420 in the p@Y527 inhibited enzyme, has been described for other systems [79]. The scaling of NMDAR function was computed as the ratio of membrane NMDAR over total NMDAR (Fig. S1D).
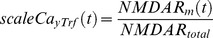
(2)
- Src-like mediated enhancement of single channel activity (ySCh): DA triggered tyrosine phosphorylation of NMDAR can also have a fast cAMP-independent Src-dependent enhancing effect on NMDAR Ca2+ currents [19]. It is known that tyrosine phosphorylation by Src-like kinase can enhance single-channel NMDAR function [80] by increasing the opening probability with STEP producing the reverse effect [81]. The residues responsible for these effects have not been pointed out. We model this mechanism taking advantage of the submodel developed by the Fyn/STEP regulation of NMDAR traffic but in this case the scaling factor (scaleCa_y_) was computed similarly to the fast modulation by PKA/PP1 (Eq 1), i.e. as a function of the ratio between the NMDAR phosphorylated in the membrane over total NMDAR in the membrane,
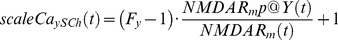
(3)where Fy is the fold increase in conductance upon Src phosphorylation but in practice it was set to have an effect whose amplitude is of similar size than the other two mechanisms (see above) for comparison of the time courses.

#### Crosstalk between the axes

There are several instances of bidirectional information flow between the AC5 and the NMDAR axes. Like in the previous model [23], Ca^+2^ from the NMDAR axis inhibits AC5 to about half of its activity and activates PP2A and PP2B which dephosphorylate D32p@75 and D32p@34, respectively. In the opposite direction, the increase of PKA/PP1 activity ratio in the AC5 axis results in an increase of the phosphorylation of the NR1 NMDAR subunit which has been linked to stronger single channel currents [62]–[64]. The increase in the PKA/PP1 activity ratio has also been claimed to inhibit STEP, a tyrosine phosphatase which counteract the activation of Fyn, NMDAR and ERK in the NMDAR axis [26].

PKA has been found to phosphorylate STEP in the KIM (Kinase Interaction Motif) region [53], and this is counteracted by PP1 [82]. The phosphorylation level of STEP has also been found to be reduced in cultures of striatal neurons treated with high concentration of NMDA (100 uM), an effect that is prevented by treatments with inhibitors of PP2B so that it is probably mediated by the de-inhibition of PP1 through the dephosphorylation of D32p34 by PP2B [82], [83]. There is a body of experimental findings that with a varying degree of extrapolation suggest that this phosphorylation renders STEP less active against its substrates ERK [53], Fyn [76] and NR2B [26] (see Discussion). If phosphorylation by PKA does alter the activity of STEP on any of these substrates, this would constitute a *PKA-sensitive* crosstalk communicating dopaminergic activity sensed through the AC5 axis to the NMDAR axis as it has been found a sizable change in the phosphorylation state of STEP upon dopamine stimulation [12]. For the analysis of the contribution of these purportedly PKA-sensitive crosstalking edges via STEP to the dynamics of the system, we considered all combinations between three factors which were expressed using a three membered binary vector: PKA-sensitive crosstalk via STEP at the level of Fyn and NR2B (0 no, 1 yes), PKA-sensitive crosstalk via STEP at the level of ERK (0 no, 1 yes) and the number of STEP pools acting at these two points (0 one, 1 two) (Table S1). For example, 010 means no crosstalk at Fyn&NR2B, crosstalk at ERK and a single shared STEP pool operating at both points. The Fyn and NR2B nodes were lumped together to reduce the number of combination and because they both control the level of NR2B phosphorylation. A second naming scheme was used to graphically identify the crosstalking scheme. In this case, each crosstalking edge is numbered and a crosstalking scheme is identified by a sequence of numbers representing the edges involved (Table S1). The number of STEP pools involved was considered an important factor in the face of sequestration effects or retroactivity [84]. That is, if the STEP acting on different substrates comes from a single pool, the sequestration in the form of Michaelis-Menten complex by one substrate affects the availability of STEP to others. In the simulations, the implementation of each crosstalking scheme was achieved by selectively turning on/off the forward rate constant of the interaction of the phosphorylated substrates with different forms/pools of STEP (Supporting Information, Table S1).

#### D1R/Golf signaling compartments

One of the most consequential modifications introduced to the previous model is that D1R and Golf are distributed in two signaling compartments with slow redistribution between them: an AC5 axis-linked compartment and a NMDAR axis-linked compartment. This redistribution occurs through a non-signaling reservoir. The distribution of D1R and Golf each of the two compartments is determined by the affinity and the total amounts (or capacity) of anchors or adaptors for these molecules present in each compartment. There are several lines of evidence motivating this assumption (see Discussion). The estimation of the amounts and affinities of the anchors in each compartment for D1R and Golf is an optimization problem. The optimal values should be such that, without being extreme, allow the model to reproduce the phenotypic data. This problem has multiple solutions and we show one obtained by manual fitting. For example, we assumed the capacity of the AC5-compartment to be at most 20% of the total amount of D1R in the system, just as the total D1R in the *Drd1a^+/−^* is 20% of the total D1R in the WT. Furthermore, the affinity for D1R in this compartment (or more precisely the ratio between the amount of anchor and its affinity for D1R) is higher than in the NMDAR compartment. This implies that in the WT the AC5 compartment will be saturated of D1R. Furthermore, in the competition between both compartments for D1R, the AC5 compartment will prevail over the NMDAR comportment up to the point that in *Drd1a^+/−^* mice the amount of D1R in the AC5 compartment will be at WT levels (because the anchor was set to 20% of WT D1R and the affinity is high) while the NMDAR compartment will be almost emptied of D1R. Interestingly, there is evidence of a D1R reserve in D1R+MSN [85] measured. An earlier study of D1R levels in the striatum of mice with a single copy of *Drd1a^+/−^* reported 40% of the WT [86] instead of the 20% used in this work. As the results about phosphorylation of GluR1 and ERK in *Drd1a*^+/−^ mice were obtained in a C57B1/6 background [19], [20] where a 20% of WT D1R level was measured we have chosen this number over the 40% measured in the hybrid 129 and C57B1/6 background [86]. We have used this 40% in a second version of the model and the conclusions of this work remained unaltered (see below).

The distribution of Golf is just the opposite of D1R: it is loosely attached to the AC5 compartment but strongly bound to the NMDAR compartment where the amount of its anchor is at most 40% of the total amount of Golf in the WT.

### Target phenotypes

The model was constrained with several quantitative molecular phenotypes gathered from the literature (Table 1). These phenotypes can be classified as APA and non-APA phenotypes depending on whether they were obtained with the APA protocol. There are 10 phenotypes several of them with more than one phenotypic variable. The names of the phenotypic variables result from the merge of the phenotype name and the marker name.

Some of these phenotypic variables are measurements of protein markers by immunohistochemistry and/or immunoblotting. Whenever possible, the measured values of protein levels by immunoblotting were corrected if, i) the samples have a relevant cellular heterogeneity such as those from the striatum, but ii) the effect is cell specific and iii) other cells types in the sample contribute to the basal levels of the protein of interest. The correction performed was,
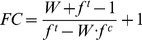
(4)FC is the cell-specific fold change over the basal, W is the fold change quantified with Western blots in the heterogeneous sample and f^c^ and f^t^ are the fraction of positive cells for the marker as measured by immunohistochemistry for the control and the treatment, respectively. The derivation of this equation is presented in Text S1 (Supporting Information).

Time series and dose-response phenotypic data was compressed by parameterization with the fitting of simple parametric models. For example, the dose-response data of phenotypes activeRAS and sensitizedNMDAR was parameterized with a Hill model.
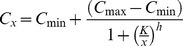
(5)where *C_min_* and *C_max_* are the minimal and maximal levels of the response (*C_x_*), respectively. *K* and *h* are the dosage for half of the total response change and the Hill coefficient. In the case of *sensitizedNMDAR*, where two dose-response data series were fitted, it is reported the ratio between the half-activating concentrations without and with dopamine.

The time series of *trafficNMDAR* was parameterized with a single exponential.
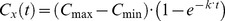
(6)where *k* is the rate constant.

Thus, the quality of the fit of the ODE model to the experimental data was represented and evaluated in these cases through the comparison of optimal parameters for the experimental data versus those of the simulated model. No simple parametric model was found to fit the time series of APAib and they were represented independently. After these transformations and excluding the phenotype ‘sensitizedNMDAR’ which was matched just qualitatively, there are a total of 17 phenotypic variables to constrain the model.

#### Model simulation and input functions

The reactions in the model were divided into three non-overlapping groups: i) enzymatic reactions, ii) reversible reactions and iii) irreversible reactions, which include transport reactions.
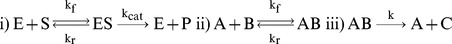


In all cases, the reaction rate was expressed according to the law of mass action. No steady-state approximation was used for any species. The resulting system of ODEs was set and solved deterministically with the ode15s solver in the SimBiology environment, a MatLab toolbox. Values of rate constants were taken from the literature whenever possible, either directly (mainly k_cat_ for enzymatic reactions) or constrained by published dissociation constants for binding reactions (K_d_ = k_r_/k_f_) and Michaelis constants (K_M_ = (k_cat_+k_r_)/k_f_, k_r_ = 4•k_cat_) for enzymatic reactions. However, very little data of this kind is found in the literature and then the quantitative molecular phenotypes are the most important source of constrains for some critical rate constants. Most of the rate constants, either first order (s^−1^) or second order (uM^−1^ s^−1^), are in the range 10^−3^ to 10^3^ in the model. The lower bound of this range was further reduced in highly lumped sections of the model, like the ERK-DUSP negative feedback where transport to the nucleus, transcription and translation are lumped. Similarly, the total amounts of the species in the network were taken from the literature whenever possible (Supporting Information, Table S2).

For the simulation of APA phenotypes, the Ca^2+^ signal is modeled as random Poisson spike train with a rate of 0.1 s^−1^ and the psychostimulant-induced dopamine as a transient increase in this neuromodulator. Both a Ca^2+^ spike and the transient overflow of dopamine are modeled by a sum of exponentials [87],
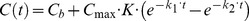
(7)
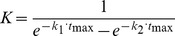
(8)
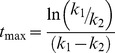
(9)where C(t) is the concentration in time, C_b_ is the basal concentration and C_max_ is the maximum amplitude of the transient.

In the case of NMDAR-mediated Ca^2+^ spikes, Ca_b_ = 60 nM, Ca_max_ = 500 nM, k_1_ = 17.2 s^−1^ and k_2_ = 15.7 s^−1^ [88].

The simulated time course of GluR1 and ERK activation upon APA was noisy due to the random Ca^2+^ spike train and the mean of replicated runs converged to the simulated time course obtained with a train of regularly spaced Ca^2+^ spikes with the same frequency (0.1 s^−1^). The difference between the mean of 20 simulated ERK activation time-courses with the random Ca^+2^ spike trains as input and the one obtained with a single train of regularly spaced Ca^2+^ spikes has an R^2^ = 0.995. Thus, this regular Ca^2+^ spike train was used in all cases unless stated otherwise.

The time constants of the dopamine transient are a consensus of different measurements of psychostimulant induced DA overflow in the striatum : C^11^-cocaine levels in the brain after i.v. administration in humans, the DA mediated psychostimulant effects follows the same kinetics [89]; nomifensine-evoked DA measured by FSCV (Fast Scanning Cyclic Voltammetry)[90] and amplitude of electrically evoked DA by FSCV after cocaine administration. Eq.7 fit these three experimental datasets with r^2^>0.96 in all cases. Thus, DA_b_ = 10 nM [91], DA_max_ = 300 nM, k_1_ = 0.15 min^−1^ and k_2_ = 0.055 min^−1^.

### Parameter sensitivity

Considerable system insights can be retrieved by analyzing how the output (corresponding to different phenotypic variables) depends on the model parameters [92]. Both when it comes to how robust the model is in terms of parameter variations [93], [94], as well as mapping out which parameters that have the largest influence on the model output [95]. It has for example been observed that biological models tend to have a “sloppy” spectra of parameter sensitivities [96], meaning that there are many parameters that has minor or no effect on the model output when perturbed slightly. Other studies have shown that different model outputs can have different groups of parameters that are the most influential to the behavior [95], [97]. In order to investigate these features in our model we performed a local sensitivity analysis, calculating normalized sensitivities *S_ij_*. This corresponds to investigating what effect minor perturbations of the parameter values have on the different model outputs. The local sensitivity of the output *o_i_* with respect to the parameter *p_j_*, was given by
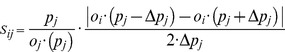
(10)which (for small enough values of Δ*p_j_*) is an approximation of the normalized partial derivative 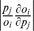 calculated at *p_j_*. Here we used a relative perturbation size of 1%, i.e. 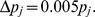 The retrieved sensitivities 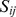 was used to consider a number of model-features; i) the sensitivity profile of the output (the phenotypic variables), e. g. which phenotypic variables are in general most sensitive to perturbations, ii) the sensitivity profile of the parameters, e.g. how influential are the different parameters on the different outputs, iii) the similarity (or dissimilarity) of the subgroups, consisting of the most sensitive parameters, for each phenotypic variable. The comparison between subgroups of sensitive parameters was done by considering all pairs of phenotypic variables. For each pair we calculated the overlap between the respective sensitive parameters, i.e. the number of sensitive parameters that the two phenotypic variables have in common. This was next divided by the number of sensitive parameters in the larger of the two subgroups to get a relative measure. This means that totally overlapping groups have an overlapping measure of one, whereas non-overlapping groups get zero.

Even though during the modelling process the enzymatic reactions was modelled directly with mass action kinetics with no steady-state assumption (using forward and backward rate constants k_f_, k_r_ and catalytic efficiency k_cat_) for the sensitivity analysis only k_cat_ and Michaelis constant K_M_ were considered. This was motivated from the realization that for enzymatic reactions, k_f_ and k_r_ could be changed considerably, but as long as the relation was kept so that K_M_ ( = (k_r_+k_cat_)/k_f_) was not changed this had no effect on the model fitting (Supporting Information, Fig. S2). K_M_ was perturbed through k_f_ (i.e. a perturbation of k_f_ with the factor (1±0.005)^−1^) and when k_cat_ was perturbed we introduced a balancing change in k_f_ and k_r_ to keep K_M_ constant so that the relevance of k_cat_ were probed independent of its contribution to K_M_ (i.e. when perturbing k_cat_ with a factor 1±0.005, k_f_ and k_r_ were also both perturbed with the same factor).

Overall, each of the 316 parameters (all 276 kinetic rate constants, as well as the total amounts of all 40 species) was varied one-at-a-time and each of the 17 different phenotypic variables was recorded.

## Results

### Building and constraining the model

The final version of the model developed in this work contains 235 reactions involving 184 species and 358 reaction rate parameters (Fig. 1 and Fig. S1). This constitutes a near 3 fold expansion with respect to a previous version [23]. The model was manually constrained to a relative large collection of molecular phenotypic data obtained from the literature. These data come from in vivo, slices and cell culture experiments and most of them correspond to D1R+MSN phenotypes but there is also some from unspecified MSNs, prefrontal cortex and hippocampal neurons. The data from striatal slices either after in vivo treatment (such as the APA protocol) or treatment of the slices themselves were corrected to account for cell type-specific response (Equation 4, Table 2).

**Table 2:** Correction of fold-changes immunoblot estimates of marker phosphorylation in D1R+MSNs (See Text S1 for details).

Treatment	Effector	Reported	Corrected
5′ after D1R agonist on striatal slices	D32p34	6 X	11 X
15′ after APA	STEPp	2.6X	4.2X
	ERKpp	2.2X	30 X
	GluR1p845	6X	11X

The validity of this simple correction is illustrated through the correspondence between the estimated fold-change for D32p34 in the DAsliceD32 phenotype (11×) and the value measured experimentally with a novel cell-type specific technique (12×) [6]. This is relevant because the capacity to sequester PP1 is doubled, thus generating a larger boost to PKA-mediated phosphorylation. However, while this procedure alleviates the underestimation of fold changes characteristic of immunoblot measurements of cell-type specific events in heterogeneous samples, the reported data seldom have all the elements required for the estimation. For example, an important part of the experiments with mutant mice lacks immunohistochemistry analysis so that estimations are not possible in a simple way. Thus, when comparing the activation of relevant effectors upon APA in mutant versus wild type mice, the ratio of changes in the APA relative to saline was used.

**Figure 2:**
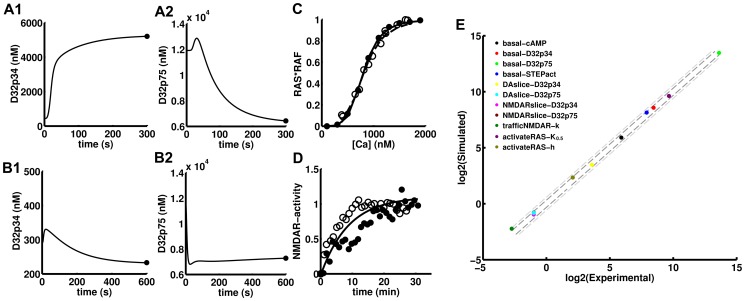
Fitting of the model to non-APA (Acute Psychostimulant Administration) phenotypes (see Table 1). Experimental values are represented with dots from A to D. A) D32p34 (A1) and D32p75 (A2) in the DAsliceD32 phenotype (sampled at 5′). B) D32p34 (B1) and D32p75 (B2) in the NMDAsliceD32 phenotype (sampled at 10′). C) Dose response of the RAS*RAF complex relative to total RAS in the activateRAS phenotype. The Hill equation was fitted to experimental data (○, dashed line) and simulated data (•, solid line) were fitted to by the Hill equation (dash line) and the Hill coefficient compared. D) Kinetics of NMDAR function enhancement in the trafficNMDAR phenotype. NR2B traffic was induced by DA in prefrontal cortex slices (•) and ethanol in the striatum (○). The mean rate constant and amplitude of a monoexponential fit are reported. The maximum amplitude produced by this mechanism has been found to lie between 1.5 for EPSPs amplitude [60] and about 4 for mEPSPs frequency [61]. E) Simulated versus experimental single time measurements and parameter estimates for all non-APA phenotypes. The dashed lines delimit 20% (dark grey) and 50% (light grey) divergence between experimental and simulated values.

The quality of fit of the model to non-APA phenotypes for both single time/dose measurements (Fig. 2A and 2B) and time-series/dose-response (Fig. 2C and 2D) was very high with divergences below 20% in most cases (Fig. 2E). The parameterization of time-series and dose-response sets with a monoexponential (Eq. 6) and the Hill equation (Eq. 5), respectively, was successful for both experimental and simulated data (r^2^>0.95) (Fig. 2C and 2D).

**Figure 3:**
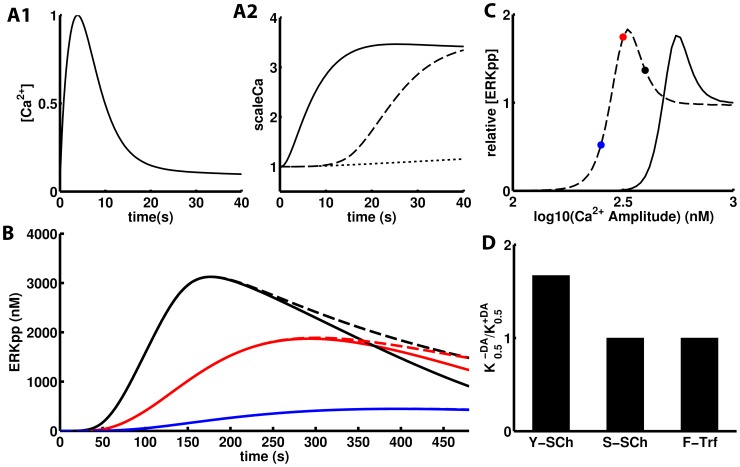
Sensitization of NMDAR Ca^2+^ triggered ERK activation by dopamine in cultured MSNs. A1) Unitary ERK activating Ca^2+^ transient whose amplitude is scaled up by DA-triggered signaling. A2) DA-induced Ca^2+^-scaling factors through different mechanism: single channel enhancement via tyrosine phosphorylation (ySCh, solid), single channel enhancement via serine/threonine phosphorylation by PKA (sSCh, dashed) and traffic based enhancement by Fyn phosphorylation of NR2B subunits (yTrf, dotted). B) Time course of ERK activation at three different Ca^2+^ pulse amplitudes in the presence of dopamine. In the dashed curves, the ERK-DUSP negative feedback was turned off. The colors correspond to the dots in panel C. C) The dose-response curve of Ca^2+^ triggered ERK activation at 8 minutes after dopamine (3 uM) addition (10 minutes in the experimental data) (dashed) is left-shifted relative to the control with no dopamine (solid) for the ySCh mechamism. In both curves ERKpp was normalized relative to the value at 1 uM of Ca^2+^ amplitude ([ERKpp] = 650 nM). D) Dopamine-triggered sensitization for the three mechanisms expressed as the ratio of the half activating Ca^2+^ without over with dopamine. Just the ySCh mechanism is sensitizing in these conditions as the other are too slow to boost the early Ca^2+^ transient.

The remaining non-APA phenotype, *sensitizedNMDAR* [19], was reproduced qualitatively by the model (Fig. 3). The model shows that a fast rise in dopamine concentration does produce a sudden sensitization of the NMDAR response as measured by the increase of the scaling factor of the Ca^2+^ level. This results in the left-shift of the dose-response curve for ERK activation (Fig. 3). In the model, this increase in the scaling factor operates on a simulated Ca^2+^ elevation that reproduces the one described in experiments (Fig. 3A1) [19] making it more effective activating ERK. Comparing the three mechanisms of DA/D1R triggered enhancement of NMDAR-Ca^2+^ currents, the sensitization was seen for single channel enhancement triggered by tyrosine phosphorylation and not for PKA-mediated single channel or Fyn-mediated traffic-based enhancements as these two were too slow to match the Ca^2+^ pulse (Fig. 3A1 and A2). Similar to what is seen in the experimental data, the dose-response curves for measurements at 8 minutes show a non-monotonic behavior (Fig. 3C) [19]. The maximum ERK activation at different Ca^2+^ amplitudes shows a typical monotonic behavior, but the higher the level of the maximum the faster the decay and then the time courses intersect and produce the non-monotonic at longer times (Fig. 3B and 3C). The removal of the negative-feedback loop (DUSP-mediated) partially uncouples maximum ERK activation levels from faster decays thus displacing the intersection of time courses curved toward longer times (Fig. 3B). The model also qualitatively reproduced another result obtained in cultures of striatal neurons treated with high concentration of glutamate (100 uM), this is, the inhibition of PP2B with Cyclosporin A increased the level of phosphorylated STEP and reduced the activation of ERK (Fig. S3) [83].

### The acute psychostimulant administration paradigm. Input signals and monitored effectors

**Figure 4:**
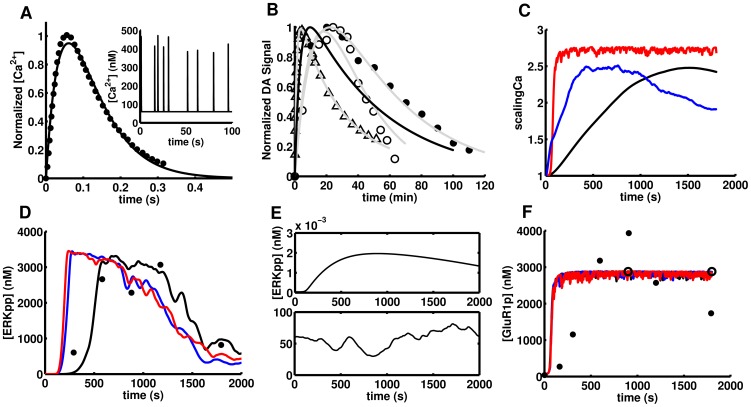
Inputs and outputs in the acute psychostimulant administration paradigm. A) A synaptically evoked Ca^2+^ spike in a single dendritic spine as measured with a fluorescent Ca^2+^ indicator [88]. Inset: 100 seconds of simulated random Ca^2+^ spikes. B) Different measurements of psychostimulant induced DA overflow in the striatum: C^11^-cocaine levels in the brain after i.v. administration in humans (Δ), the DA mediated psychostimulant effects follows the same kinetics [89]; nomifensine-evoked DA measured by FSCV [90] (○) and amplitude of electrically evoked DA by FSCV after cocaine administration (•). Eq.7 fit these three experimental datasets with r^2^>0.96 in all cases (solid grey). The solid black curve is the psychostimulant-induced DA overflow used in this work. C) Time course of the scaling factor for the three NMDAR enhancement mechanisms upon APA: the single channel mechanisms via serine/threonine (red) or tyrosine phosphorylation (blue) and the traffic based mechanism (black). D) Just the traffic-based mechanism of scaling fits the ERK activation data (r^2^ = 0.87). E) ERK is not significantly activated either by DA in the absence of Ca^2+^ spikes (upper panel) or Ca^2+^ spikes with no dopamine increase (lower panel) showing its capability as an AND gate. F) Simulated phosphorylation of GluR1 by PKA and two experimental data sets from APA-treated mice, one for methamphetamine (10 mg/Kg, •) [12] and the other for cocaine (20 mg/Kg, ○) [13]. The time course of GluR1 phosphorylation shows no dependence on the scaling mechanism. The time course of GluR1 and ERK phosphorylation was simulated with a single random Ca^2+^ spike train.

The psychostimulant induced dopamine increase in the striatum was modeled according to experimental measurements. While most of the measurements of dopamine in this and other paradigms have been performed from the extracellular fluid recovered in a microdialysis cannula, this technique underestimates both amplitude and rate [90], [98]. From a collection of several measurements made with faster and less invasive techniques, such as PET and FSCV which were fitted to a sum of exponentials (Eq. 7), a consensus psychostimulant induced DA transient with k_1_ = 0.15 min^−1^ and k_2_ = 0.055 min^−1^ was used (Fig. 4B). The Ca^2+^ input was modeled as a random Poisson train of transients with a frequency of 0.1 Hz (Fig. 4A, inset) but in most simulations a train of regularly spaced Ca^2+^ spikes with the same frequency was used in order to reduce the computational cost associated with several replicate runs. Each Ca^2+^ transient resembles the form of synaptically evoked Ca^2+^ elevation measured in hippocampal neurons (Fig. 4A)[88].

Then, the model was challenged with the DA increase generated by APA together with the Ca^2+^ spike train whose basal amplitude of 500 nM was scaled up with each of the three modeled mechanisms of DA-triggered NMDAR function enhancement: single channel enhancement through serine/threonine phosphorylation by PKA or tyrosine phosphorylation by Src-like kinases, and Fyn-mediated traffic based enhancement (Fig. 4C). The traffic-based mechanism matches very closely what has been measured experimentally in immunoblots from APA-treated mice (r^2^ = 0.87) (Fig. 4D) but the two single channel mechanisms reach maximum ERK activation far earlier than what has been reported experimentally with this technique [12]. Importantly, both dopamine increase and NMDAR Ca^2+^ entry are required for the activation of ERK (Fig. 4E), reproducing previous findings of ERK as an AND gate which is opened (activated) upon APA by the convergence of both inputs [12]. In the case of GluR1 phosphorylation there were no differences between the three mechanisms in the model, but the goodness of fit to the experimental data varies (Fig. 4F). For both effectors, the experimental data was transformed by removing its reference to the baseline levels and scaling the result so just the kinetics of the process is considered in the comparison with the simulation.

Almost all the phenotypes challenging the model so far were also reproduced by a model with a single D1R/Golf signaling compartment and a single pool PKA-sensitive crosstalk via STEP at the three nodes in the NMDAR axis (Fig. 1). However, this model failed to reproduce the activation pattern of GluR1 and ERK upon APA in D32KO, *Drd1a*^+/−^ and *GnaI^+/−^* mice as described below (Fig. S4).

### Phosphorylation of GluR1 and ERK upon APA in the D1R/Golf compartments model. Probing PKA-sensitive STEP-mediated crosstalking schemes

**Figure 5:**
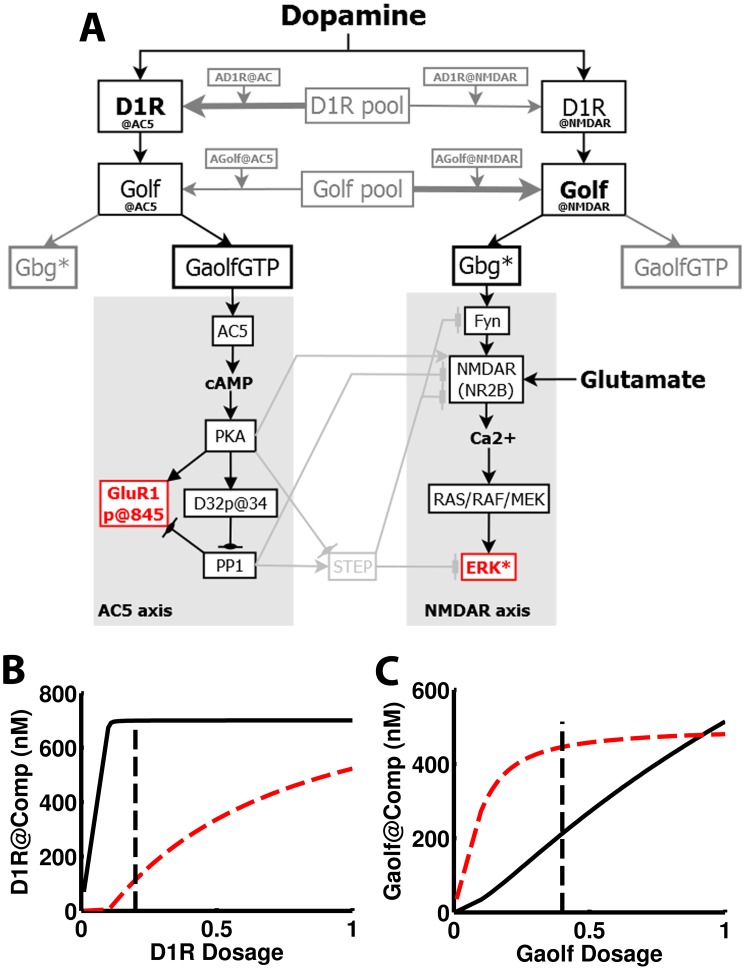
The effect of gene dosage on the distribution of D1R and Golf in the AC5 and NMDAR compartment. It was assumed that changes in Gαolf generate proportional variations in the amount of functional Golf heteromer. A) Representation of the two D1R/Golf signaling compartments, one signaling the AC5 axis and the other the NMDAR axis. Panels B and C show the distribution of D1R and Golf in the AC5 (black) and NMDAR (red) compartments, respectively, as a function of its dosage. At haploinsufficient levels (dotted line) D1R has near wild type level in the AC5 compartment (black line), while Golf is almost unaffected in the NMDAR compartment (red line). However, D1R in the NMDAR and Golf in the AC5 compartments are severely affected. This distribution is independent of the crosstalking scheme.

**Figure 6:**
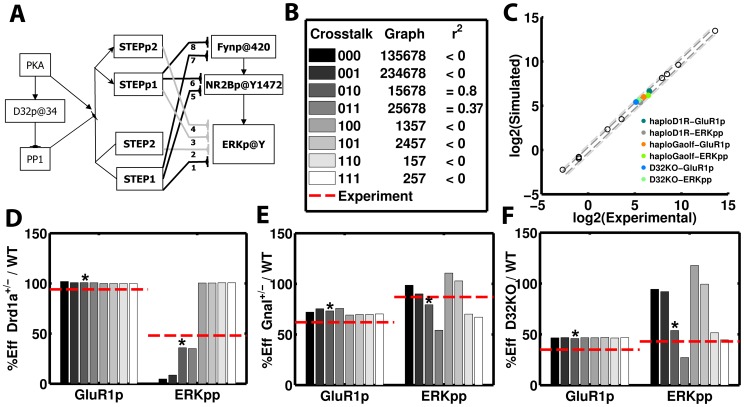
The effect of mutants on APA-induced ERK activation and GluR1 phosphorylation @845. A) Subnetwork representing the edges used to generate different crosstalking schemes. A sequence of edge numeric identifiers defines a crosstalking scheme. B) Grey-scale legend, binary encoding, edge-encoding and goodness of fit (r^2^) for the different crosstalking schemes. The resulting levels of active ERK and GluR1@845 at 15′ after psychostimulant injection do depend on the crosstalking scheme. The scheme 010 is the one closest (r^2^ = 0.8, * label) to the experimental values (dashed red line) for D1R (D) and Golf (E) haploinsufficiency and also for DARPP32 knock out (F). The other crosstalking schemes failed in one or more phenotypes as judged by the low r^2^ (B). With the 010 crosstalking scheme, the model closely matches all the phenotypes (C). The colored dots correspond to the mutant's phenotypes, while the empty circles represent the non-APA phenotypes.

The haploinsufficiency of D1R and Gαolf and the effects of D32 knock-out on the psychostimulant induced phosphorylation of GluR1 and ERK has been studied experimentally with quantitative detail [12], [19], [20]. In order to explain the opposing patterns of activation of GluR1 and ERK in haploinsufficient mice for D1R and Gαolf, we have assumed that the total pool of these elements is distributed in two compartments for D1R/Golf mediated signaling (Fig. 5A) with the amount of Golf (heterotrimer) equal to the amount of Gαolf. One of these compartments is associated with the production of cAMP via AC5 and the other with the Fyn-mediated increase of NMDAR Ca^2+^ currents. The distribution of the total amounts of D1R and Golf in each of these compartments depends on anchors that differ in affinity and capacity for D1R and Gαolf. Haploinsufficiency was simulated by reducing the total amount of D1R or Gαolf to the fraction reported experimentally (0.2 and 0.4 respectively [20]), then equilibrating the system and finally running the simulation of the APA paradigm. This was performed for each of the three mutants for 8 different PKA-sensitive STEP-mediated crosstalking schemes considered (Fig. 6A and B) in order to evaluate which of them can accommodate the existence of segregation. All these crosstalking schemes were successful reproducing the non-APA phenotypes and the APA induced transient activation of ERK and GluR1 in the wild type. They also reproduced changes in GluR1 phosphorylation for the mutants. However, there were clear differences between them in the activation level of ERK in the mutants and just the scheme with a single STEP pool mediating the PKA-sensitive crosstalk just at the level of ERK (010) was able to reproduce all mutant phenotypes (Fig. 6). The rest failed in one or more of the phenotypes and we did not find any parameter values to circumvent this.

In the case of *Drd1a*^+/−^, the amount of D1R in the AC5 compartment was at wild type levels but the amount in the NMDAR compartment was significantly reduced (Fig. 5B). Upon simulated APA, model versions with PKA-sensitive crosstalk via STEP at Fyn and NMDAR (1**) did not show reductions in ERK activation (Fig. 6D). This crosstalking scheme is the one where just the non-phosphorylated form of STEP is active against Fyn and NMDAR (i.e. edges 5 and 7 but not 6 and 8 in Fig. 6A). In the case of *GnaI^+/−^*, the amount of Golf in the NMDAR compartment was at wild type levels but the amount in the AC5 compartment was significantly reduced (Fig. 5C). Upon simulated APA, there was a reduction in GluR1 phosphorylation in all schemes but this reduced flow in the AC5 axis affected the activation of ERK in all schemes with crosstalk at the level of ERK (*1*) (i.e. edges 1 and/or 3 but not 2 and/or 4 in Fig. 6A) except the one with a single STEP pool (010) (i.e. edges 15678 in Fig. 6A). The schemes with no crosstalk at all (00*) (i.e. all forms of STEP are equally active on each substrate) and with crosstalk just at Fyn and NMDAR (10*) did not show reduction in ERK activation (Fig. 6E). For the remaining mutant, D32KO, where the phosphorylation of both GluR1 and ERK has been reported to be significantly affected (Fig. 6F) the schemes with crosstalk at ERK (*1*) reproduced the reduction in ERK activation, while the rest were not affected (Fig. 6F).

All the results presented so far can also be reproduced with an alternative version of the model where some assumptions were varied in order to meet some reported values (Fig. S5). Considering that there are at least 25 GPCR in D1R+MSN which are annotated to signal via Gs, as estimated from the IUPHAR receptor database [99] and a D1R+MSN transcriptome [100], it seems likely that Golf and possibly also D1R [101] are coupled to other signaling partners as well. Thus, in this alternative version of the model it was explicitly introduced a third compartment. Furthermore, the total amount of Golf that was set to 10 times the total amount of D1R [16], [85], [102], [103] and a level of D1R in *Drd1a*^+/−^ is 40% that of the WT [86].

### Sensitivity analysis

The rate constants and the total amount of model species (conserved moieties) in the updated model (two D1R/Golf compartments and crosstalking scheme 010) were perturbed ±0.5% and for each perturbation the value of the 17 phenotypic variables was recorded.

**Figure 7:**
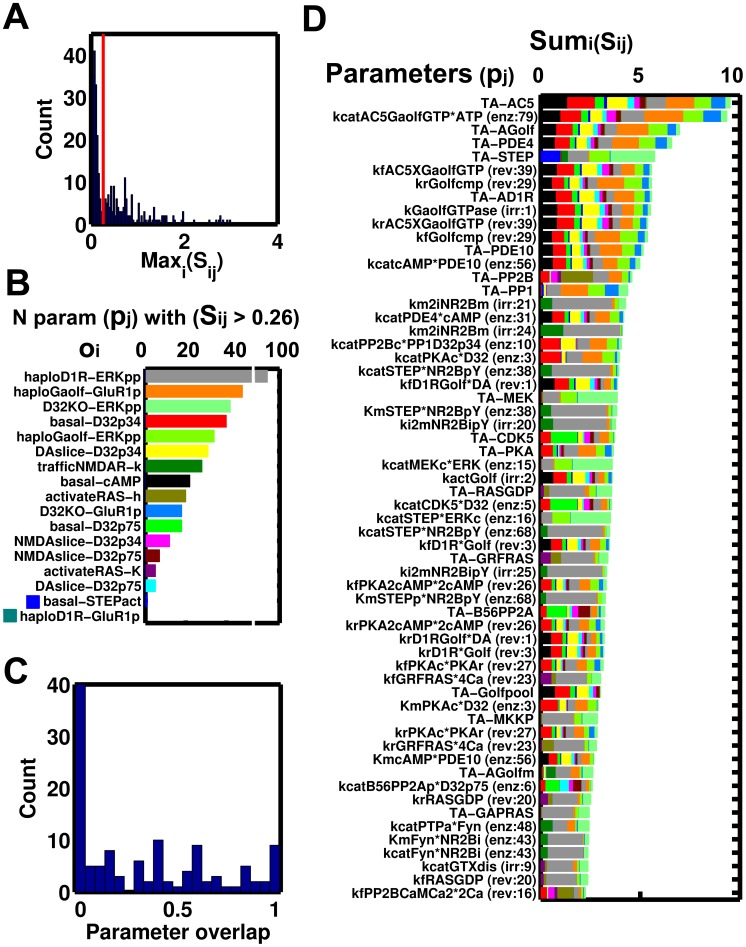
Sensitivity analysis. Reaction parameters and total amounts were considered together. A) Distribution of the maximum sensitivity Max_i_(S_ij_) of all parameters p_j_, where the maximum for each parameter is taken across all phenotypic variables o_i_. The red line indicates the distribution median (0.26). B) Number of sensitive parameters (S_ij_>0.26) for each phenotypic variable o_i_. C) Distribution of the overlap of sensitive parameters in pairs of phenotypic variables. The overlap was calculated as the number of common sensitive parameters between the two phenotypic variables divided by the number of sensitive parameters in the phenotypic variable with the highest number of sensitive parameters. D) Ranking of parameters p_j_ based on the sum of their sensitivities Sum_i_(S_ij_) across all phenotypic variables o_i_. Just the 60 parameters with the highest sensitivity scores are listed. The color code for each phenotypic variable is the one used in B. TA stands for total amount.

The distribution of the maximum value of the sensitivity to each parameter across all phenotypic variables appeared multimodal (Fig. 7A). Considering the median of this distribution (0.26) as the demarcation between insensitive and sensitive parameters, it was clear that each phenotypic variable was sensitive to a varying number of parameters (Fig. 7B). The activation of ERK in *Drd1a*^+/−^ mice upon APA was the most sensitive phenotypic variable with twice as many parameters with a large influence on the phenotype as the next in rank. On the other hand, the phosphorylation of GluR1 in the same conditions was not sensitive to any parameter. The phenotypic variables were compared pairwise (Fig. 7C) to evaluate the extent of overlap of sensitive parameters. The fraction of sensitive parameters that phenotypic variable pairs had in common covered all the range from 0 to 1 (Fig. 7C). There were several pairs of phenotypic variables with no sensitive parameters in common (e.g. trafficNMDAR-k and DAslice-D32p34) and a few pairs where the sensitive parameters of one of the phenotypic variables could all be found among the sensitive parameters of the other phenotypic variable (e.g. D32KO-GluR1 and D32KO-ERKpp). Thus, as described for quantitative signaling models for other cell types and signaling pathways [95], the parameter sensitivity profile depends on which phenotypic variable that are observed. As an example of this, phenotypic variables referring to the same marker (e.g. ERK) measured for different mutants have significantly different number and identity of sensitive parameters. In particular, we have yet to explore the origin of hypersensitivity of the activation of ERK in *Drd1a*^+/−^ mice upon APA (haploD1R-ERKpp).

Considering the detailed sensitivity profile of the parameters (Fig. 7D), total amounts (conserved moieties) are significantly enriched in the first 60 most globally sensitive parameters (50%, p<10^−10^). The total amount of AC5 and the k_cat_ of its Ca^2+^-free, Golf activated form are the two most sensitive parameters, being around 25% higher than the following in the rank (Fig. 7D). These two parameters as well as many others within the 60 most sensitive parameters are mostly related to generation and degradation of cAMP, in correspondence with the widespread effects of this second messenger in the modeled network.

In accordance with earlier studies of parameter sensitivities [96] we also find that a large part of the parameters have only a minute effect on the output. Looking at all combinations of parameters and phenotypic variables, only 8.2% of these correspond to a parameter that have a sensitive effect on a phenotypic variable (using the same threshold as earlier S_ij_ = 0.26). If the average effect that a parameter has on the phenotypic variables is considered then 4.4% of the parameters have an average sensitivity higher than the threshold (i.e. 
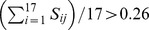
).

Here we have performed a local sensitivity analysis, using small perturbations. In studies of model robustness, larger parts of the parameter space are explored [94], [97], as well as the effect of perturbing combination of parameters. This kind of study is out of the scope of this article. We have however, within this study, tried to use a bit larger perturbations (±20%). Interestingly enough, looking at the top 20 sensitive parameters and comparing ±20% to ±0.5% perturbations, the identity of the parameters are almost the same (data not shown).

## Discussion

In this work we have developed a quantitative signaling model for striatal D1R+MSN comprising two inputs, dopamine and glutamate, operating through D1R and NMDAR receptors and regulating the activation of GluR1 and ERK. The model is constrained by a relatively large amount of phenotypic data. Furthermore, this model provides a mechanistic explanation for patterns in the data which have not been interpreted so far.

### ERK as an AND gate

Neither the Ca^2+^ spike train alone nor the psychostimulant induced dopamine in the absence of NMDAR Ca^2+^ spikes are able to activate ERK in the simulations. This matches the experimental observations of ERK as an AND gate which is opened (activated) upon APA by the convergence of glutamatergic *AND* dopaminergic inputs [12], [104]. The D1R-triggered signaling operates on both ends of the MAPK cascade [12], [19]. Upstream, D1R activation triggers the enhancement of NMDAR mediated Ca^2+^ currents. In the simulations, just the D1R-triggered traffic-based mechanism of NMDAR enhancement generates a time course of APA-induced ERK activation that matches the immunoblot-based observations. The single-channel mechanisms operate too fast. A faster APA-induced ERK activation has been measured by immunohistochemistry [8] which is more in line with the single-channel mechanism. However, the activation is expressed as the percentage of positive cells and this will clearly produce a faster kinetics than immunoblots as the latter track total levels of active ERK. On the other hand, in vivo measurements with Ca^+2^ indicators in D1R+MSNs upon APA have shown an increase in intracellular Ca^2+^ levels that matches the time scale of the slow traffic-based mechanism (Fig. 4D) [105]. Thus, the fast D1R-triggered sensitization of NMDAR to glutamate seen for MSNs in culture [19] may not be the one underlying the transient activation of ERK upon APA.

If a slow acting mechanism of ERK activation underlies the development of conditioned place preference, this may impose a minimum required residence time of the animal in the drug-paired site just after the injection for the correct association to be established. In this regard, it is worth noting that previous models of signaling triggered by convergent glutamatergic and dopaminergic inputs on D1R+MSN have failed to reproduce the effective time windows between conditioning stimulus and reward in reinforcement learning paradigms [23].

ERK has been reported to be activated in cultures of striatal neurons treated only with a D1R agonist (SKF81297) [106]. This effect was sensitive to PKA and Src inhibition and involves the tyrosine phosphatase Shp-2 which forms a complex with D1R. However, the activation level of ERK achieved with D1R agonist alone is significantly lower than the level obtained in co-treatment with NMDAR agonists [19] in similar cultures which in turns operates in a cAMP-independent way but also relies on a Src-like kinase Fyn. We did not include in our model this NMDAR-independent activation of ERK by D1R agonists because still little is known about the mechanism of this interesting possibility which was suggested by the authors to be location dependent in the striatum [106]. Furthermore, its contribution to ERK activation is partial and the potential role of background glutamate has not been probed with, for example, NMDAR antagonists. On the other hand, the cAMP-independence of ERK activation upon co-stimulation with D1R and NMDAR in these cultures [19] seems contradictory under the light of the conclusions of this paper. However, the phosphorylation level of STEP in these cultures in basal conditions is rather high [83] so that D1R stimulation has little room to operate through this locus and is then mostly confined to the sensitization of glutamatergic input through NMDAR [19].

### Parameter sensitivities

In order to evaluate how the model output (the phenotypic variables) depend on the parameters (reaction rates, total amounts) a local sensitivity analysis was performed. In accordance with earlier studies [96], this model displays a wide profile of sensitivities where a large part of the parameters have very low or almost no effect on the output when perturbed locally. We further noted that the sensitivity profile depended a lot on which of the outputs that were observed, with some pairs of phenotypic variables having no sensitive parameters in common at all. This feature, that the sensitivity depend on the outputs that are monitored, has also been observed in models for other cell types [95], [97]. Finally, the sensitivity analysis provided new intriguing questions about the system, the most important being the large sensitivity of the phenotype corresponding to activation of ERK in *Drd1a*^+/−^ mice upon APA, which we have to consider further.

### The distribution of D1R/Golf in two signaling compartments explains the segregation of effects in D1R and Gαolf haploinsufficient mice

The model with a single D1R/Golf signaling compartment reproduces the non-APA phenotypes as well as the APA paradigm time course data (APAib phenotype) in wild type animals. However, despite several fitting attempts this model fails to explain the opposed sensitivities of GluR1 and ERK phosphorylation under the APA paradigm to reductions in the total amounts of D1R and Gαolf seen in haploinsufficient animals. This pattern is in turn explained by assuming the existence of two D1R/Golf signaling compartments, one coupled to cAMP production by AC5 and the other to the enhancement of NMDAR-mediated Ca^2+^ currents with both connected through a non-signaling pool. The compartmentalization of GPCR/G-protein signaling has been suggested before for explaining diverse experimental observations [107]. In the case discussed here, this compartmentalization is a necessary but insufficient condition. It should be complemented with a differential affinity and capacity of the anchors in each compartment for D1R and Gαolf. That is, while D1R is strongly bound to the AC5 compartment it is loosely attached to the NMDAR compartment and the opposite holds for Golf. In this way it is explained that a decrease in the total amount of D1R preferentially affects the amount of D1R in the NMDAR compartment while reductions in Gαolf preferentially affect the content Golf in the AC5 compartment. The sensitivity of the cAMP signaling to reductions of 60% in Golf but not of 80% in D1R total amounts is puzzling since it has been measured that the amount of Golf in MSNs is more than 10X higher that D1R [16], [85], [102], [103]. Similarly curious is that in Parkinsonian patients and animal models of Parkinson disease, Golf is upregulated while D1R is not [108]. These estimates lack spatial resolution so that it is not known which fraction of both D1R and Golf is present in the plasma membrane. We used similar total amounts of D1R and Golf in this model because even if this 10X ratio were kept in the plasma membrane, a significant portion of Gαolf may be pre-coupled to several other Gs-coupled GPCR provided that D1R+MSN have negligible amounts of Gαs [18]. Thus, it is significant that the conclusions of this work were the same with an alternative version of the model (Fig. S5) where i) the existence of D1R and Golf coupled to other signaling partners is considered explicitly with the introduction of a third compartment, ii) the total Golf is 10 times the total D1R and iii) the level of D1R in *Drd1a*^+/−^ is 40% that of the WT.

The nature of the AC5-linked compartment is possibly a cholesterol rich membrane domain containing caveolin-1, a cholesterol binding protein known to interact with D1R [29] and expressed in striatal MSNs [30]. AC5 has been found to be enriched in lipid rafts in several systems and it also interacts with caveolin-1 [109]. The NMDAR-linked compartment is possibly located in the postsynaptic density (PSD) or its vicinity. D1R has a direct physical interaction with NR1 and NR2A subunits of NMDAR which is located preferentially in the post-synaptic density (PSD) [31]. This interaction have been found to be functional in MSNs [31], [32] and this location is a potential candidate for the D1R-mediated sensitization of NMDAR to glutamate. D1R has been also found to interact with PSD-95 [110]. Importantly, evidence of D1R distribution in these two compartments has been found in prefrontal cortex neurons, which like the striatum receive midbrain dopaminergic innervation. In these cells, D1R was found enriched in the detergent-resistant membrane fraction usually associated to lipid rafts and also in the dense fraction associated with PSD proteins [28]. The identity of the anchors of G-proteins in general and Gαolf in particular to each compartment is less clear. There are several reports of GPCR coupled to different signaling cascades depending on the compartmentalization of the receptor within a cell [111]. For example, in HEK293 cells it was found that A2aR in lipid rafts were associated to adenylyl cyclase activation while A2aR outside the rafts were linked to ERK activation [112]. The effect of reducing the total amounts of the receptor on the signaling strength of *each* signaling cascade in these systems has not been studied.

While the evidence makes plausible the segregation of the D1R/Golf pair into two compartments, there are other alternatives especially for the coupling of Golf to the D1R in the NMDAR compartment. PKC has been found by some to be mediating the DA-induced enhancement of NMDAR currents [113]. This enzyme can be activated via Gq by D1R-D2R heteromers [101] which have been found to be functional in the striatum albeit just in a reduced fraction of MSNs of adult mice [8]. There are also reports of some D1R-like agonists (e.g. SKF83959) activating Gq signaling [114].

### Are the effects of PKA-catalyzed phosphorylation of STEP substrate-dependent?

In the face of unidirectional crosstalk from the AC5 axis to the NMDAR axis, it is reasonable to expect a transmission of the sensitivity to reductions in the total amount of Gαolf from the AC5 axis to effectors in the NMDAR axis like ERK [27]. In fact, the knock-out of D32, or its T34A mutation, decreases the phosphorylation of both GluR1 and ERK upon APA showing that an insufficient increase in the PKA/PP1 activity ratio upon dopaminergic signaling do have a negative impact on ERK phosphorylation [12]. However, as mentioned before, a strong segregation of the effects of D1R and Gαolf haploinsufficiency on the phosphorylation of GluR1 and ERK has been observed experimentally. How can the PKA-sensitive crosstalk via STEP accommodate these apparently contradicting experimental observations?

The premise for the crosstalk is that just the non-phosphorylated form of STEP is able to act on the targets in the NMDAR axis. If both the phosphorylated (STEPp) and non-phosphorylated forms of STEP are catalytically equivalent toward a given target there is no crosstalk because changes in the PKA/PP1 activity ratio upon dopaminergic action have no effect on overall STEP activity toward this target. While the evidence of STEP phosphorylation by PKA upon dopaminergic stimulation is abundant [12], [24], [53], [115], there is no direct experimental evidence of the effects of this modification on the tyrosine phosphatase activity of STEPp toward each of its three targets in the NMDAR axis: Fyn, NR2B and ERK. First, STEPp has been found to have a two-fold increase of Km toward a model substrate, myelin basic protein [53], but its activity on these targets has not been measured in biochemical experiments. For ERK as substrate, it has been found that STEP dephosphorylates it [116] but the evidence of STEPp inactivity comes from homologous tyrosine phosphatases PTP-SL and HePTP that upon phosphorylation by PKA in the KIM domain lose almost all activity against ERK and/or the ERK homologous p38a [24], [25]. The dephosphorylation of STEPp by PP1 is also an extrapolation from this homologous phosphatases [82]. For Fyn it was found that its interaction with STEP is lost if the KIM domain is removed [76], but the activity of STEPp toward this substrate has not been measured. Finally, for NR2B far less is known about the activity of STEPp towards its intrinsically disordered cytoplasmatic tail where Y1472 lies [117]. Another issue of relevance is whether or not the STEP/STEPp operating on different substrates belongs to the same or different STEP pools. Sequestration effects seem to be pervasive in biochemical systems [84], [118], [119] and if the phosphatase operating on different substrates comes from a single pool, its sequestration by one substrate in the Michaelis-Menten complex would reduce the dephosphorylation pressure on the others. It has been found that substrates of ERK control the activity of this kinase against other substrates through a sequestration-based retroactive mechanism [120]. At least two variants of catalytically competent STEP have been detected [53].

Clearly, the PKA-sensitive STEP-mediated crosstalking scheme is still an open question and the reproduction of this non-trivial data pattern, i.e. segregation despite crosstalk, is an interesting challenge. The fitting of the model with different crosstalking schemes provided some insights and *one* possible solution to this conundrum. After several attempts to fit the model with each of these 8 crosstalking schemes, the scheme with a single STEP pool mediating a crosstalk just at the ERK node was successfully fitted.

With the parameters from the fitting of the model with the 010 scheme (Table S2), the model with each of the other 7 schemes was run and the fitting recorded in order to gain insight about their failure. Each of these 7 schemes failed to reproduce at least one of the APA induced ERK activation phenotypes in the mutant mice:

- In the absence of crosstalk at any level disregarding the number of STEP pools (00*), the model reproduced the segregation but then D32KO had no impact on ERK activation because there is no information flow from the AC5 axis to the NMDAR axis.
- With crosstalk at the level of Fyn&NR2B irrespective of the status at ERK and the number of STEP pools (1**), D1R haploinsufficiency did not affect the activation of ERK because the deficit in D1R/Golf mediated activation of Fyn-p@527 is balanced by the inactivation of STEP through PKA mediated phosphorylation.
- With crosstalk just at the level of ERK but 2 STEP pools (011), the segregation is affected as the activation of ERK in *Gnal^+/−^* is reduced in the same extent than GluR1 phosphorylation. This contrasts with the effectiveness of a single STEP pool in the 010 scheme. A single STEP pool is a pre-requisite for the sequestration of STEP by ERKpp to alleviate the tonic inhibition that STEP and STEPp has on NMDAR currents thus compensating the reduction in the inhibition of STEP by PKA taking place in *Gnal^+/−^* upon APA. In fact, the effectiveness of 010 is lost with a 10 fold increase in the K_M_ of STEP on ERKpp, which implies a reduction in complex formation between STEP and ERKpp and thus in the extent of the sequestration. This effect was not due to a reduction in enzymatic activity because we kept the k_cat_/K_M_ ratio constant by increasing the k_cat_.

From this we predict that the effect of the phosphorylation of STEP by PKA on its activity is substrate-dependent with only little changes for Fyn and NR2B and significant inhibition for ERK. Furthermore, the STEP/STEPp operating on the three nodes of the NMDAR axis comes from a single pool, as this enables a sequestration or retroactive compensatory mechanism that operates as a positive feedback loop. This alleviates the coupling between the axes so that the segregation of the effects of D1R and Golf haploinsufficiency is kept despite the existence of cr osstalk. The claim that the PKA-phosphorylated form of STEP is inactive [26], is based on the observations of homologous enzymes like PTP-SL and HePTP [24], [25] with ERK as substrate. The validity of this extrapolation requires further analysis.

In conclusion, with a model for the dynamics of the intracellular signaling triggered by dopamine and glutamate, which integrates a relatively high amount of quantitative data, we have been able to provide a rationale for some previously unexplained experimental results. Two critical assumptions were required: i) the existence of at least two D1R/Golf signaling compartments each of them coupled to a different cascade and ii) that the multiple STEP-mediated interactions between these cascades are differentially affected by the PKA phosphorylation of this phosphatase. The existence of compartmentalized upstream signaling modules coupled to different signaling axes with segregated functional implications opens up the possibility of designing drugs targeting these elements in a compartment specific fashion. A clear requirement for this strategy is that the pharmacological profile of the receptor differs between compartments which might be plausible provided the differing composition of each of them.

## Supporting Information

Figure S1 Other sub-networks included in the model. A) Regulation of the three phosphodiesterases. B) Nucleotide exchange and RAS inactivation. C) Activation cycle of Fyn. D) Traffic of NR2B-containing NMDAR, in red exocytosis and in blue endocytosis. Any of the four forms of NMDAR represented can be modified by the PKA/PP1 cycle.
(TIF)

Figure S2 Changes in model fitting upon 2 fold increase and decrease in the rate parameters of reversible reactions without altering binding or Michaelis-Menten constants. A) Legend for phenotypes. B) Fitting of the unperturbed model. C&D) Fitting of the model after slowing down (C) or speeding up (D) binding in all enzymatic reactions in the model while keeping Km constant. The goodness of fit was unperturbed. E&F) Fitting of the model after slowing down (E) or speeding up (F) all non-enzymatic binding reactions in the model while keeping Kd constant. In this case, when reducing the reaction rates (E) the goodness of fit to several phenotypes was significantly affected. GEF and GAP activities on Golf and RAS were not included among the enzymatic reactions.
(TIF)

Figure S3 Variations in the level of phosphorylated STEP (STEPp) and active ERK upon the treatment with high glutamate concentration (represented as tonic 10 uM of Ca2+) with (blue) and without (red) Cyclosporin A, a PP2B inhibitor [83]. The inhibition of PP2B with Cyclosporin A increases the level of inactive STEP (STEPp) allowing an increased activation of ERK by glutamate (possibly via NMDAR). Notice that the action of PP2B on STEPp is not direct, but via D32p34/PP1. The log scale of the y-axis in the graph of panel A was used to illustrate changes relative to the basal level but it should be highlighted that in the cultured cells used to generate the data being reproduced here [83] the level of phosphorylated STEP in basal conditions is rather high while in our model this level is low as it reproduces the observation made in striatal slices [12].
(TIF)

Figure S4 Fitting of a single compartment model. Not all phenotypes could be fitted. The outliers for this parameter set are identified with an arrow in the legend. The identity of the outliers can change for other parameters sets which produce a similar fitting quality, but in all cases the mutant phenotypes is the source of most of the outliers.
(TIF)

Figure S5 An alternative version of the multi-compartment model. This version has three compartments for D1R/Golf, with total Golf near ten times total D1R and the haploinsufficient level of D1R in *Drd1a*^+/−^ equal to 40%. The third compartment for D1R (X) and Golf (Y) doesn't needs to be the same as these species can be coupled to other signaling partners. Despite these differences with the model in the main text (two compartments, D1R/Golf ∼1 and 20% of D1R remaining in *Drd1a*^+/−^) the fit quality and the conclusions are the same. Compare the panels I, II and III to those in Figure 5 and panels A to F to those in Figure 6 in the main text.
(TIF)

Table S1 Generation of the 8 crosstalking schemes by turning on/off forward rate constants of the interaction between different forms and pools of STEP (phosphorylated and non-phosphorylated) and its phosphorylated substrates (Fynp, NR2Bp and ERKpp). “Fynp” and “NMDARp” represent any phosphorylated forms of active Fyn and NMDAR. STEP2 is a second pool of STEP. The crosstalking schemes are encoded in two different forms. One (first row) with a 3 member binary vector (see main text) and the other (second row) according to the numbered edges depicted in Fig. 6A of the main text.
(PDF)

Table S2 Rate parameters and total amounts. The rate parameters and total amounts were taken from the literature whenever possible, but in most cases they resulted from the manual fitting of the model to phenotypic data. Thus, most of the references in the last column of the following tables are a guide to the edges but not their weight. The reactions were color coded as a function of the sub-network they belong to.
(PDF)

Text S1 Quantifying protein marker changes in heterogeneous samples.
(PDF)
