# Supplementary figures and images for: Segregation and Crosstalk of D1 Receptor-Mediated Activation of ERK in Striatal Medium Spiny Neurons upon Acute Administration of Psychostimulants
Source: PLoS Comput Biol. 2014 Jan 30;10(1):e1003445. doi: 10.1371/journal.pcbi.1003445 (PMC3907292; doi:10.1371/journal.pcbi.1003445)

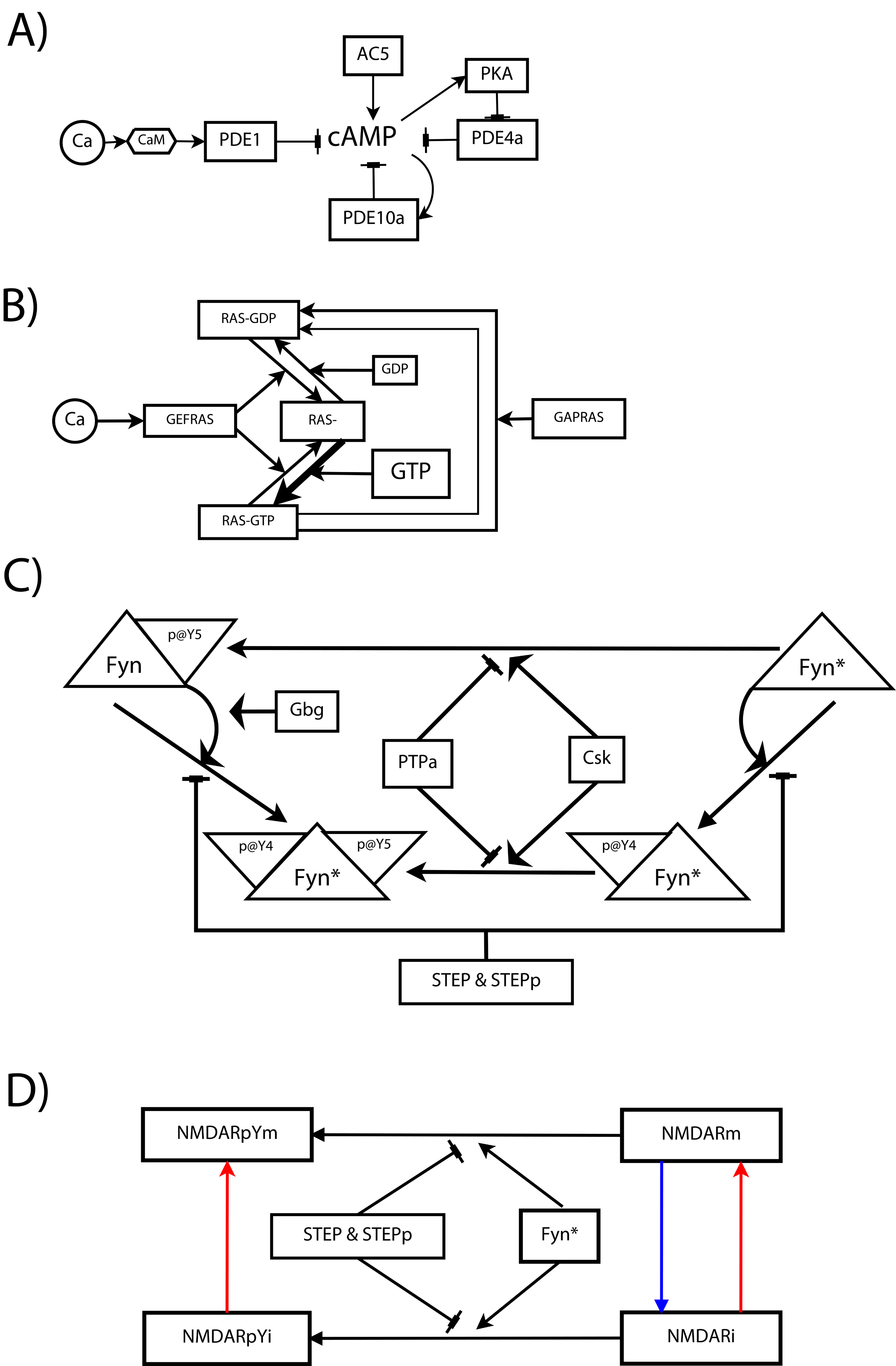

Supplement: Figure S1 — Other sub-networks included in the model. A) Regulation of the three phosphodiesterases. B) Nucleotide exchange and RAS inactivation. C) Activation cycle of Fyn. D) Traffic of NR2B-containing NMDAR, in red exocytosis and in blue endocytosis. Any of the four forms of NMDAR represented can be modified by the PKA/PP1 cycle. (TIF) [file pcbi.1003445.s001.tif]

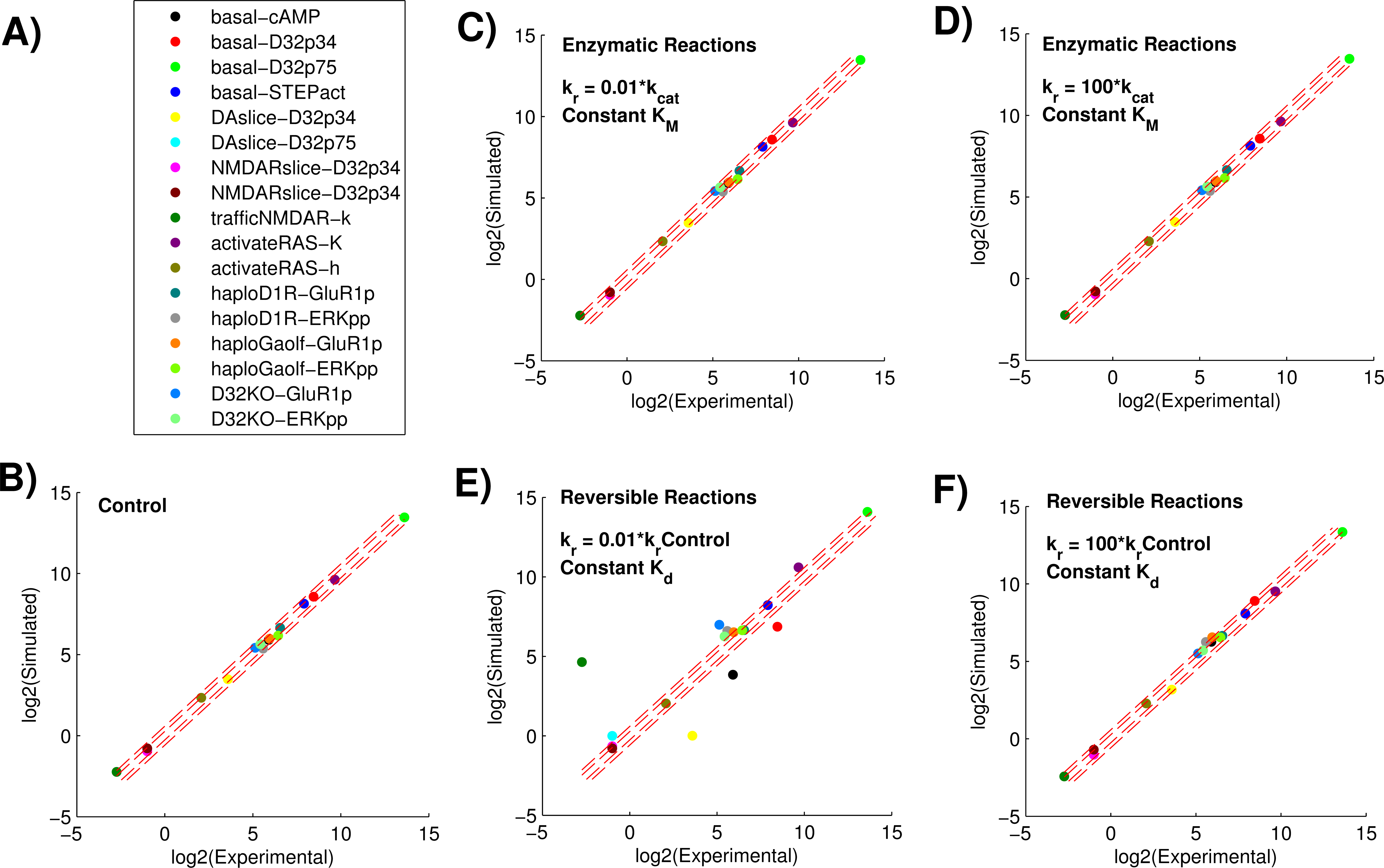

Supplement: Figure S2 — Changes in model fitting upon 2 fold increase and decrease in the rate parameters of reversible reactions without altering binding or Michaelis-Menten constants. A) Legend for phenotypes. B) Fitting of the unperturbed model. C&D) Fitting of the model after slowing down (C) or speeding up (D) binding in all enzymatic reactions in the model while keeping Km constant. The goodness of fit was unperturbed. E&F) Fitting of the model after slowing down (E) or speeding up (F) all non-enzymatic binding reactions in the model while keeping Kd constant. In this case, when reducing the reaction rates (E) the goodness of fit to several phenotypes was significantly affected. GEF and GAP activities on Golf and RAS were not included among the enzymatic reactions. (TIF) [file pcbi.1003445.s002.tif]

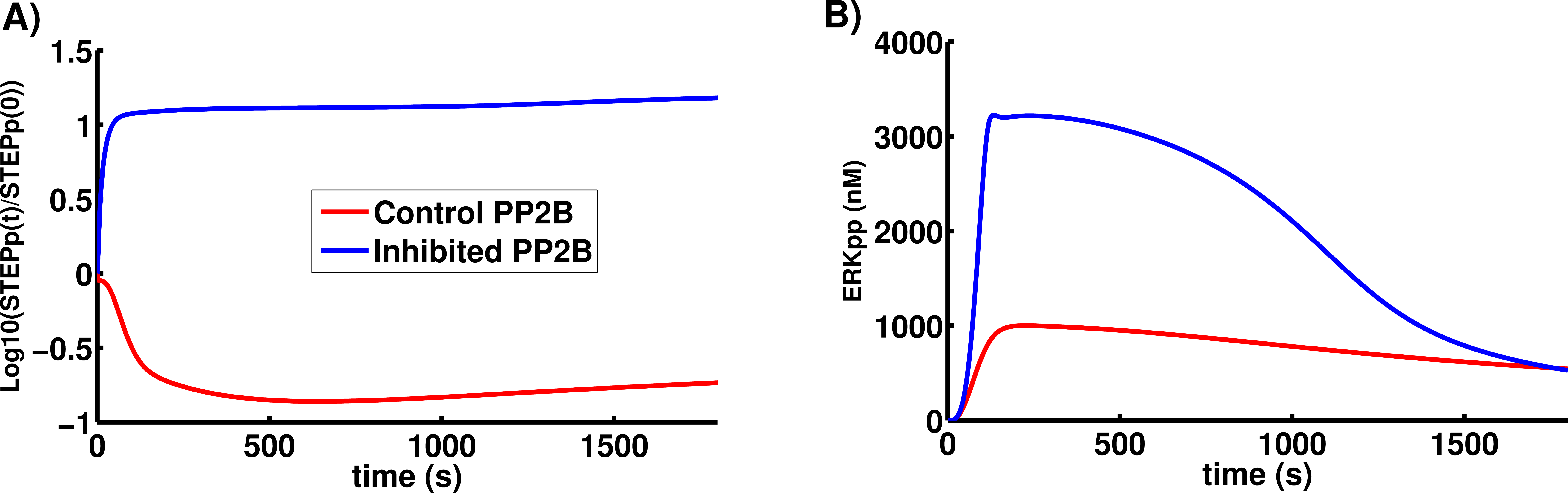

Supplement: Figure S3 — Variations in the level of phosphorylated STEP (STEPp) and active ERK upon the treatment with high glutamate concentration (represented as tonic 10 uM of Ca2+) with (blue) and without (red) Cyclosporin A, a PP2B inhibitor [83]. The inhibition of PP2B with Cyclosporin A increases the level of inactive STEP (STEPp) allowing an increased activation of ERK by glutamate (possibly via NMDAR). Notice that the action of PP2B on STEPp is not direct, but via D32p34/PP1. The log scale of the y-axis in the graph of panel A was used to illustrate changes relative to the basal level but it should be highlighted that in the cultured cells used to generate the data being reproduced here [83] the level of phosphorylated STEP in basal conditions is rather high while in our model this level is low as it reproduces the observation made in striatal slices [12]. (TIF) [file pcbi.1003445.s003.tif]

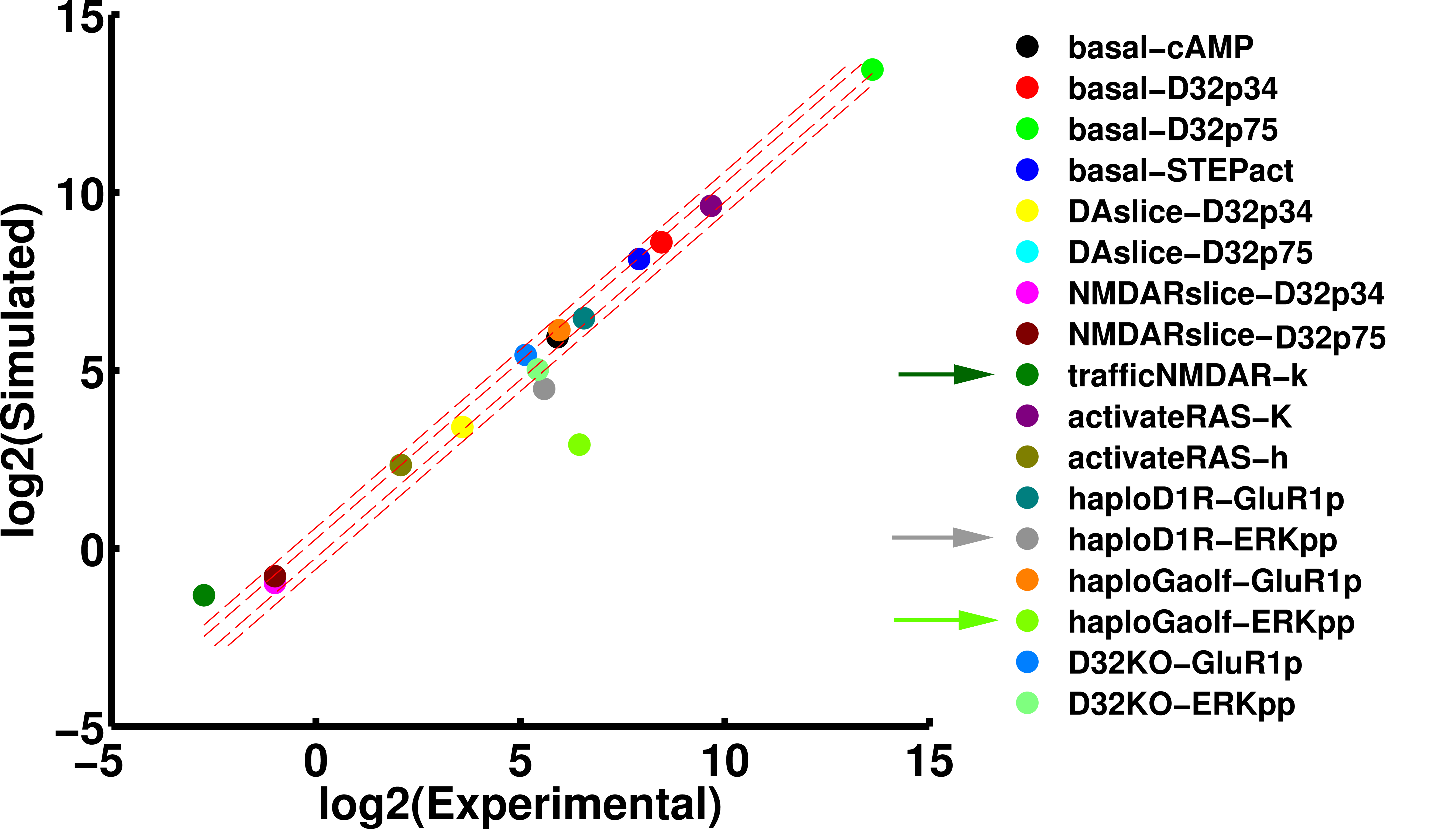

Supplement: Figure S4 — Fitting of a single compartment model. Not all phenotypes could be fitted. The outliers for this parameter set are identified with an arrow in the legend. The identity of the outliers can change for other parameters sets which produce a similar fitting quality, but in all cases the mutant phenotypes is the source of most of the outliers. (TIF) [file pcbi.1003445.s004.tif]

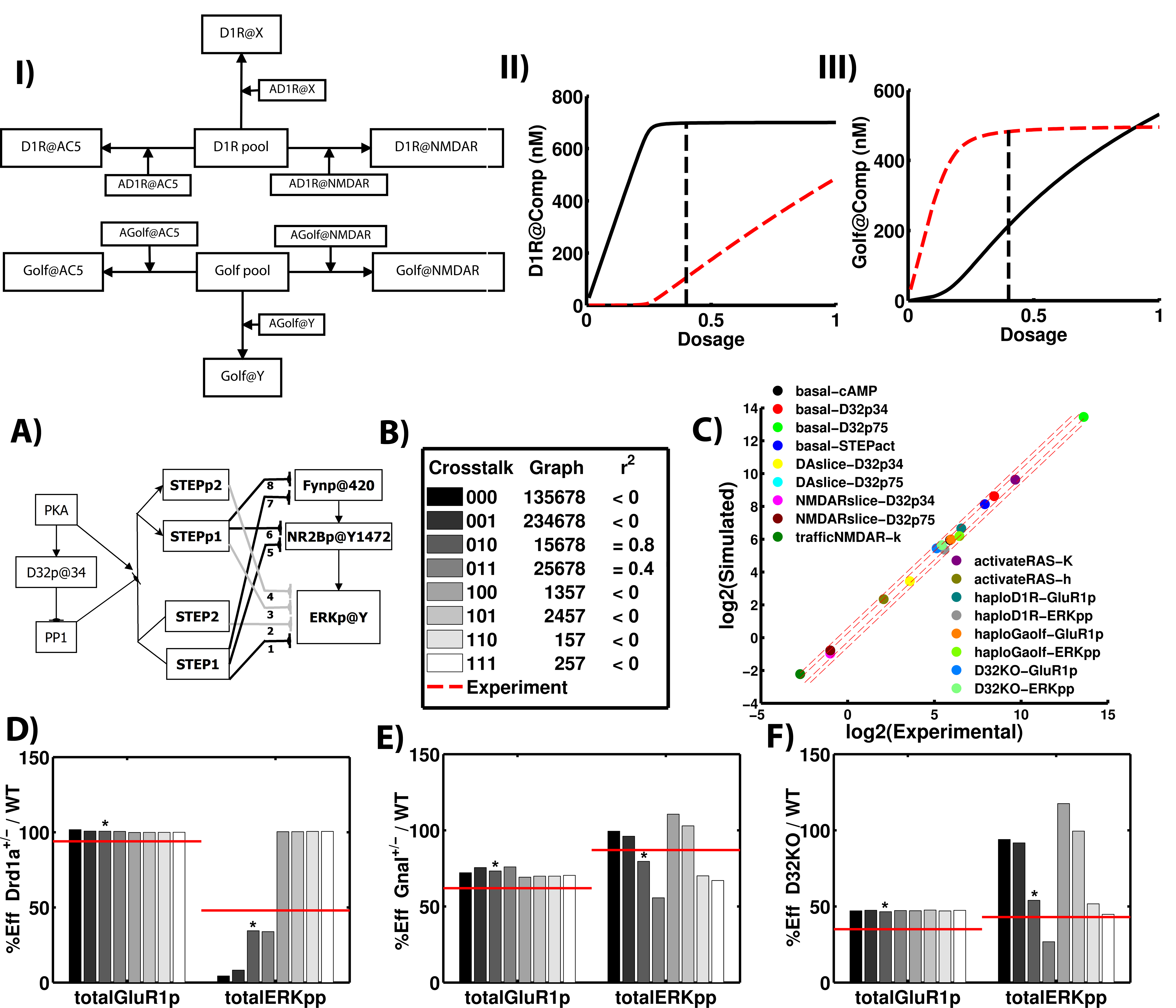

Supplement: Figure S5 — An alternative version of the multi-compartment model. This version has three compartments for D1R/Golf, with total Golf near ten times total D1R and the haploinsufficient level of D1R in Drd1a+/− equal to 40%. The third compartment for D1R (X) and Golf (Y) doesn't needs to be the same as these species can be coupled to other signaling partners. Despite these differences with the model in the main text (two compartments, D1R/Golf ∼1 and 20% of D1R remaining in Drd1a+/−) the fit quality and the conclusions are the same. Compare the panels I, II and III to those in Figure 5 and panels A to F to those in Figure 6 in the main text. (TIF) [file pcbi.1003445.s005.tif]
