# Supplementary material for: Segregation and Crosstalk of D1 Receptor-Mediated Activation of ERK in Striatal Medium Spiny Neurons upon Acute Administration of Psychostimulants
Source: PLoS Comput Biol. 2014 Jan 30;10(1):e1003445. doi: 10.1371/journal.pcbi.1003445 (PMC3907292; doi:10.1371/journal.pcbi.1003445)
Supplement: Table S1 — Generation of the 8 crosstalking schemes by turning on/off forward rate constants of the interaction between different forms and pools of STEP (phosphorylated and non-phosphorylated) and its phosphorylated substrates (Fynp, NR2Bp and ERKpp). “Fynp” and “NMDARp” represent any phosphorylated forms of active Fyn and NMDAR. STEP2 is a second pool of STEP. The crosstalking schemes are encoded in two different forms. One (first row) with a 3 member binary vector (see main text) and the other (second row) according to the numbered edges depicted in Fig. 6A of the main text. (PDF) [file pcbi.1003445.s006.pdf]

Table S1: Generating crosstalking schemes

Table SI: Generation of the 8 crosstalking schemes by turning on/off forward rate constants of the interaction between different forms and pools of STEP (phosphorylated and non-phosphorylated) and its phosphorylated substrates (Fynp, NR2Bp and ERKpp). "Fynp" and "NMDARp" represent any phosphorylated forms of active Fyn and NMDAR. STEP2 is a second pool of STEP. The crosstalking schemes are encoded in two different forms. One (first row) with a 3 member binary vector (see main text) and the other (second row) according to the numbered edges depicted in Fig. 6A of the main text.

**kf ($nM^{-1}\cdot s^{-1}$)**	**Crosstalking scheme**							
	**000**	**001**	**010**	**011**	**100**	**101**	**110**	**111**
	**135678**	**234678**	**15678**	**25678**	**1357**	**2457**	**157**	**257**
**STEP*Fynp**	0.0042	0.0042	0.0042	0.0042	0.0042	0.0042	0.0042	0.0042
**STEPp*Fynp**	0.0042	0.0042	0.0042	0.0042	0	0	0	0
**STEP*NMDARp**	0.0094	0.0094	0.0094	0.0094	0.0094	0.0094	0.0094	0.0094
**STEPp*NMDARp**	0.0094	0.0094	0.0094	0.0094	0	0	0	0
**STEP*ERKpp**	0.01	0	0.01	0	0.01	0	0.01	0
**STEPp*ERKpp**	0.01	0	0	0	0.01	0	0	0
**STEP2*ERKpp**	0	0.01	0	0.01	0	0.01	0	0.01
**STEP2p*ERKpp**	0	0.01	0	0	0	0.01	0	0
