# Supplementary material for: Segregation and Crosstalk of D1 Receptor-Mediated Activation of ERK in Striatal Medium Spiny Neurons upon Acute Administration of Psychostimulants
Source: PLoS Comput Biol. 2014 Jan 30;10(1):e1003445. doi: 10.1371/journal.pcbi.1003445 (PMC3907292; doi:10.1371/journal.pcbi.1003445)
Supplement: Table S2 — Rate parameters and total amounts. The rate parameters and total amounts were taken from the literature whenever possible, but in most cases they resulted from the manual fitting of the model to phenotypic data. Thus, most of the references in the last column of the following tables are a guide to the edges but not their weight. The reactions were color coded as a function of the sub-network they belong to. (PDF) [file pcbi.1003445.s007.pdf]

Table S2: Rate parameters and total amounts

The rate parameters and total amounts were taken from the literature whenever possible, but in most cases they resulted from the manual fitting of the model to phenotypic data. Thus, most of the references in the last column of the following tables are a guide to the edges but not their weight. The reactions were color coded as a function of the sub-network they belong to.

	**D1R and G-protein activation cycle. Compartmental distribution.**
	**Generation, hydrolysis and effectors of cAMP.**
	**D32 sub-network.**
	**Activation and inactivation of most Ca^{2+} binding proteins.**
	**PKA substrates phosphorylation and dephosphorylation, except NMDAR.**
	**Activation cycle of Fyn.**
	**Tyrosine phosphorylation, dephosphorylation and traffic of NMDAR (NR2B).**
	**Phosphorylation and dephosphorylation of NMDAR (NR1) by PKA.**
	**MAPK cascade and DUSP negative feedback.**

Enzymatic reactions

ID	Reaction	kf (nM-1s-1)	kr (s-1)	kcat (s-1)	References
enz:79	AC5GaolfGTP + ATP <-> AC5GaolfGTP*ATP -> AC5GaolfGTP + cAMP	0.006	96	24	[1–5]
enz:80	AC5 + ATP <-> AC5*ATP -> AC5 + cAMP	0.00025	4	1	[1–5]
enz:81	AC5CaGaolfGTP + ATP <-> AC5CaGaolfGTP*ATP -> AC5CaGaolfGTP + cAMP	0.003	48	12	[1–5]
enz:82	AC5Ca + ATP <-> AC5Ca*ATP -> AC5Ca + cAMP	0.000125	2	0.5	[1–5]
enz:1	PDE1c + cAMP <-> PDE1c*cAMP -> PDE1c + AMP	0.0046	44	11	[1]
enz:2	PDE4p + cAMP <-> PDE4p*cAMP -> PDE4p + AMP	0.25	40	10	[1]
enz:31	PDE4 + cAMP <-> PDE4*cAMP -> PDE4 + AMP	0.083	8	2	[6]
enz:30	PKAc + PDE4 <-> PKAc*PDE4 -> PKAc + PDE4p	0.014	40	10	[6]
enz:58	PDE4PPase + PDE4p <-> PDE4PPase*PDE4p -> PDE4PPase + PDE4	0.033	8	2	[6]
enz:56	PDE10 + cAMP <-> PDE10*cAMP -> PDE10 + AMP	0.083	8	2	[7–9]
enz:57	PDE10c + cAMP <-> PDE10c*cAMP -> PDE10c + AMP	0.5	40	10	[7–9]
enz:3	PKAc + D32 <-> PKAc*D32 -> PKAc + D32p34	0.0047	28	7	[1]
enz:9	PP2Bc + D32p34 <-> PP2Bc*D32p34 -> PP2Bc + D32	0.021	6	1.5	[1]
enz:10	PP2Bc + PP1D32p34 <-> PP2Bc*PP1D32p34 -> PP2Bc + PP1 + D32	0.017	4	1	[1]
enz:11	B72PP2A + D32p34 <-> B72PP2A*D32p34 -> B72PP2A + D32	0.0024	0.4	0.1	[1]
enz:12	B72PP2A + PP1D32p34 <-> B72PP2A*PP1D32p34 -> B72PP2A + PP1 + D32	0.000062	0.4	0.1	[1]
enz:24	B72PP2ACa + D32p34 <-> B72PP2ACa*D32p34 -> B72PP2ACa + D32	0.0024	0.4	0.1	[1]
enz:25	B72PP2ACa + PP1D32p34 <-> B72PP2ACa*PP1D32p34 -> B72PP2ACa + PP1 + D32	0.000062	0.4	0.1	[1]
enz:5	CDK5 + D32 <-> CDK5*D32 -> CDK5 + D32p75	0.0011	8	2	[1]
enz:23	B56PP2A + D32p75 <-> B56PP2A*D32p75 -> B56PP2A + D32	0.00047	6	1.5	[1,10]
enz:6	B56PP2Ap + D32p75 <-> B56PP2Ap*D32p75 -> B56PP2Ap + D32	0.002	32	8	[1,10]
enz:7	B72PP2A + D32p75 <-> B72PP2A*D32p75 -> B72PP2A + D32	0.000068	4	1	[11,12]
enz:8	B72PP2ACa + D32p75 <-> B72PP2ACa*D32p75 -> B72PP2ACa + D32	0.0017	24	6	[11,12]
enz:77	PP1 + CaMKIIc <-> PP1*CaMKIIc -> PP1 + CaMCa4CaMKII	0.000039	0.34	0.086	[12]
enz:78	PP1 + CaMKIIp <-> PP1*CaMKIIp -> PP1 + CaMKII	0.000039	0.34	0.086	[12]
enz:14	PKAc + STEP <-> PKAc*STEP -> PKAc + STEPp	0.014	40	10	[13]
enz:13	PP1 + STEPp <-> PP1*STEPp -> PP1 + STEP	0.0029	32	8	[14]
enz:83	PKAc + STEP2 <-> PKAc*STEP2 -> PKAc + STEP2p	0.014	40	10	[13]
enz:84	PP1 + STEP2p <-> PP1*STEP2p -> PP1 + STEP2	0.0029	32	8	[14]
enz:54	PKAc + GluR1 <-> PKAc*GluR1 -> PKAc + GluR1p	0.08	40	10	[12]
enz:55	PP1 + GluR1p <-> PP1*GluR1p -> PP1 + GluR1	0.035	9.6	2.4	[12]
enz:4	PKAc + B56PP2A <-> PKAc*B56PP2A -> PKAc + B56PP2Ap	0.0013	0.4	0.1	[1,10]
enz:47	STEP + Fynpa <-> STEP*Fynpa -> STEP + Fyn	0.0042	4	1	[15]
**enz:51**	**STEPp + Fynpa <-> STEPp*Fynpa -> STEPp + Fyn**	0.0042	4	1	[15]
enz:39	FynpiGolf + FynpiGolf <-> FynpiGolf*FynpiGolf -> FynpapiGolf + FynpiGolf	0.083	0.8	0.2	[16–19]
enz:36	STEP + Fynpapi <-> STEP*Fynpapi -> STEP + Fynpi	0.0042	4	1	[15]

**enz:50**	**STEPp + Fynpapi <-> STEPp*Fynpapi -> STEPp + Fynpi**	0.0042	4	1	[15]
enz:40	Csk + Fyn <-> Csk*Fyn -> Csk + Fynpi	0.13	20	5	[16–18]
enz:48	PTPa + Fynpi <-> PTPa*Fynpi -> PTPa + Fyn	0.00096	0.8	0.2	[16–18]
enz:41	Csk + Fynpa <-> Csk*Fynpa -> Csk + Fynpapi	0.13	20	5	[16–18]
enz:49	PTPa + Fynpapi <-> PTPa*Fynpapi -> PTPa + Fynpa	0.00096	0.8	0.2	[16–18]
enz:43	Fyn + NMDARi <-> Fyn*NMDARi -> Fyn + NMDARipY	0.017	40	10	[16,20,21]
enz:45	Fynpa + NMDARi <-> Fynpa*NMDARi -> Fynpa + NMDARipY	0.017	40	10	[16,20,21]
enz:34	Fynpapi + NMDARi <-> Fynpapi*NMDARi -> Fynpapi + NMDARipY	0.017	40	10	[16,20,21]
enz:38	STEP + NMDARipY <-> STEP*NMDARipY -> STEP + NMDARi	0.0094	12	3	[22,23]
**enz:52**	**STEPp + NMDARipY <-> STEPp*NMDARipY -> STEPp + NMDARi**	0.0094	12	3	[22,23]
enz:44	Fyn + NMDARm <-> Fyn*NMDARm -> Fyn + NMDARmpY	0.017	40	10	[16,20,21]
enz:46	Fynpa + NMDARm <-> Fynpa*NMDARm -> Fynpa + NMDARmpY	0.017	40	10	[16,20,21]
enz:35	Fynpapi + NMDARm <-> Fynpapi*NMDARm -> Fynpapi + NMDARmpY	0.017	40	10	[16,20,21]
enz:37	STEP + NMDARmpY <-> STEP*NMDARmpY -> STEP + NMDARm	0.0094	12	3	[22,23]
**enz:53**	**STEPp + NMDARmpY <-> STEPp*NMDARmpY -> STEPp + NMDARm**	0.0094	12	3	[22,23]
enz:69	Fyn + NMDARipS <-> Fyn*NMDARipS -> Fyn + NMDARipYpS	0.017	40	10	[16,20,21]
enz:70	Fynpa + NMDARipS <-> Fynpa*NMDARipS -> Fynpa + NMDARipYpS	0.017	40	10	[16,20,21]
enz:71	Fynpapi + NMDARipS <-> Fynpapi*NMDARipS -> Fynpapi + NMDARipYpS	0.017	40	10	[16,20,21]
enz:67	STEP + NMDARipYpS <-> STEP*NMDARipYpS -> STEP + NMDARipS	0.0094	12	3	[22,23]
**enz:68**	**STEPp + NMDARipYpS <-> STEPp*NMDARipYpS -> STEPp + NMDARipS**	0.0094	12	3	[22,23]
enz:74	Fyn + NMDARmpS <-> Fyn*NMDARmpS -> Fyn + NMDARmpYpS	0.017	40	10	[16,20,21]
enz:73	Fynpa + NMDARmpS <-> Fynpa*NMDARmpS -> Fynpa + NMDARmpYpS	0.017	40	10	[16,20,21]
enz:72	Fynpapi + NMDARmpS <-> Fynpapi*NMDARmpS -> Fynpapi + NMDARmpYpS	0.017	40	10	[16,20,21]
enz:75	STEP + NMDARmpYpS <-> STEP*NMDARmpYpS -> STEP + NMDARmpS	0.0094	12	3	[22,23]
**enz:76**	**STEPp + NMDARmpYpS <-> STEPp*NMDARmpYpS -> STEPp + NMDARmpS**	0.0094	12	3	[22,23]
enz:59	PKAc + NMDARm <-> PKAc*NMDARm -> PKAc + NMDARmpS	0.017	8	2	[24]
enz:60	PP1 + NMDARmpS <-> PP1*NMDARmpS -> PP1 + NMDARm	0.017	8	2	[25]
enz:61	PKAc + NMDARmpY <-> PKAc*NMDARmpY -> PKAc + NMDARmpYpS	0.017	8	2	[24]
enz:62	PP1 + NMDARmpYpS <-> PP1*NMDARmpYpS -> PP1 + NMDARmpY	0.017	8	2	[25]
enz:64	PKAc + NMDARi <-> PKAc*NMDARi -> PKAc + NMDARipS	0.017	8	2	[24]
enz:63	PP1 + NMDARipS <-> PP1*NMDARipS -> PP1 + NMDARi	0.017	8	2	[25]
enz:66	PKAc + NMDARipY <-> PKAc*NMDARipY -> PKAc + NMDARipYpS	0.017	8	2	[24]
enz:65	PP1 + NMDARipYpS <-> PP1*NMDARipYpS -> PP1 + NMDARipY	0.017	8	2	[25]
enz:22	RASGTP + GAPRAS <-> GAPRAS*RASGTP -> GAPRAS + RASGDP	0.00089	0.36	0.09	[26–28]
enz:29	RAFc + GAPRAS <-> GAPRAS*RAFc -> GAPRAS + RAF + RASGDP	0.00089	0.36	0.09	[26–28]
enz:19	RAFc + MEKp <-> RAFc*MEKp -> RAFc + MEKc	0.0094	1.2	0.3	[29,30]
enz:18	MKKP + MEKp <-> MKKP*MEKp -> MKKP + MEK	0.0019	24	6	[29,30]
enz:20	RAFc + MEK <-> RAFc*MEK -> RAFc + MEKp	0.0094	1.2	0.3	[29,30]
enz:17	MKKP + MEKc <-> MKKP*MEKc -> MKKP + MEKp	0.0019	24	6	[29,30]
enz:21	MEKc + ERK <-> MEKc*ERK -> MEKc + ERKpT	0.032	1.2	0.3	[29,30]
enz:28	DUSP1 + ERKpT <-> DUSP1*ERKpT -> DUSP1 + ERK	0.0011	4	1	[29,30]
enz:32	B56PP2A + ERKpT <-> B56PP2A*ERKpT -> B56PP2A + ERK	0.000005	0.04	0.01	[31,32]
enz:33	B56PP2Ap + ERKpT <-> B56PP2Ap*ERKpT -> B56PP2Ap + ERK	0.000005	0.04	0.01	[31,32]
enz:15	MEKc + ERKpT <-> MEKc*ERKpT -> MEKc + ERKpp	0.032	1.2	0.3	[29,30]
enz:27	DUSP1 + ERKpp <-> DUSP1*ERKpp -> DUSP1 + ERKpT	0.0011	4	1	[29,30]
enz:16	STEP + ERKpp <-> STEP*ERKpp -> STEP + ERKpT	0.01	2.4	0.6	[13,32]
**enz:87**	**STEPp + ERKpp <-> STEPp*ERKpp -> STEPp + ERKpT**	0	2.4	0.6	[13,32]
**enz:85**	**STEP2 + ERKpp <-> STEP2*ERKpp -> ERKpT + STEP2**	0	2.4	0.6	[13,32]
**enz:86**	**STEP2p + ERKpp <-> STEP2p*ERKpp -> STEP2p + ERKpT**	0	2.4	0.6	[13,32]
enz:26	TF + ERKpp <-> ERKc*TF -> TFc + ERKpp	0.000021	0.1	0.025	[33]

Reactions in bold are run depending of the crosstalking scheme. Table SI defines which reactions are activated in each crosstalking scheme. They are matched via the name of the Michaelis-Menten complex where Fynp and NMDARp in Table S1 represent any phosphorylated forms of active Fyn and NMDAR in SM2. The scheme 010 is the default one where none of these reactions in bold is active (kf = 0).

Reversible reactions

ID	Reaction	kf (nM-1s-1)	kr (s-1)	References
rev:4	D1R + DA <-> D1RDA	0.00025	10	[34,35]
rev:1	D1RG + DA <-> D1RDAGolf	0.005	2	[34,35]
rev:3	D1R + Golf <-> D1RG	0.0005	1	[34,35]
rev:2	D1RDA + Golf <-> D1RDAGolf	0.05	1	[34,35]
rev:31	D1Rm + DA <-> D1RmDa	0.00025	10	[34,35]
rev:33	D1RmGolf + DA <-> D1RmDaGolf	0.005	2	[34,35]
rev:32	D1Rm + Golfm <-> D1RmGolf	0.0005	1	[34,35]
rev:34	D1RmDa + Golfm <-> D1RmDaGolf	0.05	1	[34,35]
rev:28	AD1R + D1Rpool <-> D1R	0.1	0.1	-
rev:36	AD1Rm + D1Rpool <-> D1Rm	0.000002	0.01	-
rev:29	AGolf + Golfpool <-> Golf	0.000006	0.1	-
rev:35	AGolfm + Golfpool <-> Golfm	0.0005	0.1	-
rev:21	AC5 + Ca <-> AC5Ca	0.001	0.9	[1]
rev:41	AC5*ATP + Ca <-> AC5Ca*ATP	0.001	0.9	[1]
rev:5	AC5 + GaolfGTP <-> AC5GaolfGTP	0.00033	0.01	[1,36]
rev:22	AC5Ca + GaolfGTP <-> AC5CaGaolfGTP	0.00033	0.01	[1,36]
rev:39	AC5*ATP + GaolfGTP <-> AC5GaolfGTP*ATP	0.00033	0.01	[1,36]
rev:40	AC5Ca*ATP + GaolfGTP <-> AC5CaGaolfGTP*ATP	0.00033	0.01	[1,36]
rev:7	PDE1 + CaMCa4 <-> PDE1c	0.1	1	[1]
rev:37	PDE10 + 2 cAMP <-> PDE10c	0.000001 (*)	9	[7–9]
rev:25	PKA + cAMP <-> PKAcAMP2	0.00026	0.06	[1]
rev:26	PKAcAMP2 + cAMP <-> PKAcAMP4	0.00035	0.6	[1]
rev:27	PKAcAMP4 <-> PKAc + PKAreg	0.001	0.096	[1]
rev:12	D32p75 + PKAc <-> PKAcD32p75	0.00037	1	[1]
rev:11	D32p34 + PP1 <-> PP1D32p34	0.4	0.58	[1]
rev:13	B72PP2A + Ca <-> B72PPA2Ca	0.0001	0.1	[11,12]
rev:6	CaM + Ca <-> CaMCa2	0.006	9.1	[1]
rev:14	CaMCa2 + Ca <-> CaMCa4	0.1	1000	[1]
rev:8	PP2B + CaM <-> PP2BCaM	1	3	[1]
rev:9	PP2B + CaMCa4 <-> PP2Bc	1	0.3	[1]
rev:15	PP2BCaM + Ca <-> PP2BCaMCa2	0.006	0.91	[1]
rev:16	PP2BCaMCa2 + Ca <-> PP2Bc	0.1	10	[1]
rev:17	PP2B + CaMCa2 <-> PP2BCaMCa2	1	0.3	[1]
rev:10	CaMKII + CaMCa4 <-> CaMCa4CaMKII	0.00075	0.1	[1]
rev:38	CaMKIIc <-> CaMCa4 + CaMKIIp	0.013	0.0008	[1]
rev:30	Gbgolfm + Fynpi <-> FynpiGolf	0.05	5	-
rev:23	RASGRF1 + 4 Ca <-> GRFRASc	0.0000001 (**)	1000000	[26–28]
rev:18	RASGTP + RASGRF1c <-> RASGRF1cRASGTP	0.1	17	[37–39]
rev:20	RASGDP + RASGRF1c <-> RASGRF1cRASGDP	0.1	17	[37–39]
rev:19	RASGTP + RAF <-> RAFc	0.09	5	[26–28]
rev:24	2 TFc + DNA <-> TFDNA	0.01(2)	1	[33]

* $nM^{-2}s^{-1}$, ** $nM^{-4}s^{-1}$

Irreversible reactions

ID	Reaction	k (s-1)	References
irr:1	GaolfGTP -> GaolfGDP	1	[40]
irr:17	GaolfmGTP -> GaolfmGDP	1	[40]
irr:2	D1RDAGolf -> Gbgolf + D1RDA + GaolfGTP	2	[41]
irr:16	D1RmDaGolf -> D1RmDa + GaolfmGTP + Gbgolfm	2	[41]
irr:18	Gbgolfm + GaolfmGDP -> Golfm	100	[1]
irr:26	GaolfGDP + Gbgolf -> Golf	100	[1]
irr:27	cAMP + AC5GaolfGTP -> AC5GaolfGTP*ATP	0.006	[1]
irr:28	cAMP + AC5 -> AC5*ATP	0.000125	[1]
irr:29	cAMP + AC5Ca -> AC5Ca*ATP	0.00025	[1]
irr:30	cAMP + AC5CaGaolfGTP -> AC5CaGaolfGTP*ATP	0.003	[1]
irr:3	CaMCa4CaMKII -> CaMKIIc	0.005	[12]
irr:10	B56PP2Ap -> B56PP2A	0.004	[1,10]
irr:21	NMDARm -> NMDARi	0.002	[42–46]
irr:22	NMDARi -> NMDARm	0.00001	[42–46]
irr:23	NMDARipS -> NMDARmpS	0.00001	[42–46]
irr:24	NMDARmpS -> NMDARipS	0.002	[42–46]
irr:25	NMDARipYpS -> NMDARmpYpS	0.002	[42–46]
irr:19	FynpapiGolf -> Fynpapi + Gbgolfm	50	-
irr:4	RASGRF1cRAS -> RASGRF1cRASGDP	4	[37–39]
irr:5	RASGRF1cRAS -> RASGRF1cRASGTP	20	[37–39]
irr:8	RASGRF1cRASGTP -> RASGRF1cRAS	5	[37–39]
irr:9	RASGRF1cRASGDP -> RASGRF1cRAS	5	[37–39]
irr:6	RAFc -> RAF + RASGDP	0.00045	[47]
irr:7	RASGTP -> RASGDP	0.00045	[47]
irr:11	TFc -> TF	0.05	[33]
irr:12	TFDNA + Nucleotides -> TFDNA + DUSP1mRNA	0.03	[33]
irr:13	DUSP1mRNA + aa -> DUSP1 + DUSP1mRNA	0.06	[33]
irr:15	DUSP1mRNA -> Nucleotides	0.0003	[33]
irr:14	DUSP1 -> aa	0.00037	[33]

Total amounts

N	Species	Total amount
1	Ca	60
2	DA	10
3	D1Rpool	7000
4	Golfpool	5000
5	AD1R	700
6	AD1Rm	900
7	AGolf	2000
8	AGolfm	500
9	ATP	5000000
10	AC5	400
11	PDE1	3000
12	PDE4	1000
13	PDE4PPase	50
14	PDE10	1000
15	PKA	1200
16	CDK5	1800
17	DARPP32	50000
18	PP1	1000
19	B56PP2A	2000
20	B72PP2A	2000
21	PP2B	4000
22	CaM	10000
23	CaMKII	20000
24	Fyn	300
25	PTPa	300
26	Csk	50
27	STEP	300
28	STEP2	300
29	NMDAR	1000
30	GluR1	3000
31	RasGRF1	800
32	GAPRAS	300
33	RAS	500
34	RAF	400
35	MEK	500
36	MKKP	1000
37	ERK	3600
38	TF	50
39	DNA	1
40	Nucleotides	1
41	aa	1

References

1. Lindskog M, Kim M, Wikström M a, Blackwell KT, Kotaleski JH (2006) Transient calcium and dopamine increase PKA activity and DARPP-32 phosphorylation. PLoS Comput Biol 2: e119. doi:10.1371/journal.pcbi.0020119.

2. Dessauer C, Gilman A (1997) The catalytic mechanism of mammalian adenylyl cyclase. Equilibrium binding and kinetic analysis of P-site inhibition. J Biol Chem 272: 27787–27795.

3. Zalduegui A, López de Jesús M, Callado LF, Meana JJ, Sallés J (2011) Levels of Gsα((short and long)), Gα(olf) and Gβ((common)) subunits, and calcium-sensitive adenylyl cyclase isoforms (1, 5/6, 8) in post-mortem human brain caudate and cortical membranes: Comparison with rat brain membranes and potential stoichiometric rel. Neurochem Int 58: 180–189. doi:10.1016/j.neuint.2010.11.014.

4. Chen-Goodspeed M, Lukan AN, Dessauer C (2005) Modeling of Galpha(s) and Galpha(i) regulation of human type V and VI adenylyl cyclase. J Biol Chem 280: 1808–1816. doi:10.1074/jbc.M409172200.

5. Sadana R, Dascal N, Dessauer C (2009) N Terminus of Type 5 Adenylyl Cyclase Scaffolds Gs Heterotrimer. Mol Pharmacol 76: 1256–1264. doi:10.1124/mol.109.058370.

6. Murthy KS, Zhou H, Makhlouf GM (2002) PKA-dependent activation of PDE3A and PDE4 and inhibition of adenylyl cyclase V/VI in smooth muscle. Am J Physiol Cell Physiol 282: C508–17. doi:10.1152/ajpcell.00373.2001.

7. Fujishige K, Kotera J, Omori K (1999) Striatum- and testis-specific phosphodiesterase PDE10A Isolation and characterization of a rat PDE10A. Eur J Biochem 266: 1118–1127.

8. Jäger R, Russwurm C, Schwede F, Genieser H-G, Koesling D, et al. (2012) Activation of PDE10 and PDE11 phosphodiesterases. J Biol Chem 287: 1210–1219. doi:10.1074/jbc.M111.263806.

9. Nishi A, Kuroiwa M, Miller DB, O'Callaghan JP, Bateup HS, et al. (2008) Distinct roles of PDE4 and PDE10A in the regulation of cAMP/PKA signaling in the striatum. J Neurosci 28: 10460–10471. doi:10.1523/JNEUROSCI.2518-08.2008.

10. Ahn J-H, Mcavoy T, Rakhilin S V, Nishi A, Greengard P, et al. (2007) Protein kinase A activates protein phosphatase 2A by phosphorylation of the B56d subunit. PNAS 104: 2979–2984.

11. Ahn J-H, Sung JY, McAvoy T, Nishi A, Janssens V, et al. (2007) The B"-PR72 subunit mediates Ca2+-dependent dephosphorylation of DARPP-32 by protein phosphatase 2A. PNAS 104: 9876–9881.

12. Oliveira RF, Kim M, Blackwell KT (2012) Subcellular Location of PKA Controls Striatal Plasticity: Stochastic Simulations in Spiny Dendrites. PLoS Comput Biol 8: e1002383. doi:10.1371/journal.pcbi.1002383.

13. Paul S, Snyder G, Yokakura H, Picciotto M, Nairn A, et al. (2000) The Dopamine/D1 receptor mediates the phosphorylation and inactivation of the protein tyrosine phosphatase STEP via a PKA-dependent pathway. J Neurosci 20: 5630–5638.

14. Nika K, Hyunh H, Williams S, Paul S, Bottini N, et al. (2004) Haematopoietic protein tyrosine phosphatase (HePTP) phosphorylation by cAMP-dependent protein kinase in T-cells: dynamics and subcellular location. Biochem J 378: 335–342. doi:10.1042/BJ20031244.

15. Nguyen T, Liu J, Lombroso PJ (2002) Striatal Enriched Phosphatase 61 Dephosphorylates Fyn at Phosphotyrosine 420. Biochemistry 277: 24274 –24279. doi:10.1074/jbc.M111683200.

16. Sun G, Sharma AK, Budde RJA (1998) Autophosphorylation of Src and Yes blocks their inactivation by Csk phosphorylation. Oncogene 17: 1587–1595.

17. Sun G, Ramdas L, Wang W, Vinci J, Mcmurray J, et al. (2002) Effect of Autophosphorylation on the Catalytic and Regulatory Properties of Protein Tyrosine Kinase Src. Arch Biochem Biophys 397: 11–17. doi:10.1006/abbi.2001.2627.

18. Salter MW, Kalia L V (2004) Src kinases: a hub for NMDA receptor regulation. Nat Rev Neurosci 5: 317–328. doi:10.1038/nrn1368.

19. Pascoli V, Besnard A, Hervé D, Pagès C, Heck N, et al. (2010) Cyclic Adenosine Monophosphate-Independent Tyrosine Phosphorylation of NR2B Mediates Cocaine-Induced Extracellular Signal-Regulated Kinase Activation. Biol Psychiatry 69: 218–227. doi:10.1016/j.biopsych.2010.08.031.

20. Nakazawa T, Komai S, Tezuka T, Hisatsune C, Umemori H, et al. (2001) Characterization of Fyn-mediated tyrosine phosphorylation sites on GluR epsilon 2 (NR2B) subunit of the N-methyl-D-aspartate receptor. J Biol Chem 276: 693–699. doi:10.1074/jbc.M008085200.

21. Yu X, Askalan R, II GJK, Salter MW (1997) NMDA Channel Regulation by Channel-Associated Protein Tyrosine Kinase Src. Science (80-) 275: 674–678. doi:10.1126/science.275.5300.674.

22. Braithwaite SP, Adkisson M, Leung J, Nava A, Masterson B, et al. (2006) Regulation of NMDA receptor trafficking and function by striatal-enriched tyrosine phosphatase (STEP). Eur J Neurosci 23: 2847–2856. doi:10.1111/j.1460-9568.2006.04837.x.

23. Pelkey KA, Askalan R, Paul S, Kalia L V, Nguyen TH, et al. (2002) Tyrosine phosphatase STEP is a tonic brake on induction of long-term potentiation. Neuron 34: 127–138.

24. Skeberdis VA, Chevaleyre V, Lau CG, Goldberg JH, Pettit DL, et al. (2006) Protein kinase A regulates calcium permeability of NMDA receptors. Nat Neurosci 9: 501–510. doi:10.1038/nn1664.

25. Flores-Hernández J, Cepeda C, Hernández-Echeagaray E, Calvert CR, Jokel ES, et al. (2002) Dopamine enhancement of NMDA currents in dissociated medium-sized striatal neurons: role of D1 receptors and DARPP-32. J Neurophysiol 88: 3010–3020. doi:10.1152/jn.00361.2002.

26. Buhrman G, Senthil Kumar VS, Cirit M, Haugh JM, Mattos C (2011) Allosteric modulation of Ras-GTP is linked to signal transduction through RAF kinase. J Biol Chem 286: 3323–3331. doi:10.1074/jbc.M110.193854.

27. Yasuda R, Harvey CD, Zhong H, Sobczyk A, van Aelst L, et al. (2006) Supersensitive Ras activation in dendrites and spines revealed by two-photon fluorescence lifetime imaging. Nat Neurosci 9: 283–291. doi:10.1038/nn1635.

28. Harvey CD, Yasuda R, Zhong H, Svoboda K (2008) The spread of Ras activity triggered by activation of a single dendritic spine. Science 321: 136–140. doi:10.1126/science.1159675.

29. Sivakumaran S, Hariharaputran S, Mishra J, Bhalla US (2003) The Database of Quantitative Cellular Signaling: management and analysis of chemical kinetic models of signaling networks. Bioinformatics 19: 408–415. doi:10.1093/bioinformatics/btf860.

30. Ajay SM, Bhalla US (2004) A role for ERKII in synaptic pattern selectivity on the time-scale of minutes. Eur J Neurosci 20: 2671–2680. doi:10.1111/j.1460-9568.2004.03725.x.

31. Letourneux C, Rocher G, Porteu F (2006) B56-containing PP2A dephosphorylate ERK and their activity is controlled by the early gene IEX-1 and ERK. EMBO J 25: 727–738. doi:10.1038/sj.emboj.7600980.

32. Zhou B, Wang Z-X, Zhao Y, Brautigan DL, Zhang Z-Y (2002) The specificity of extracellular signal-regulated kinase 2 dephosphorylation by protein phosphatases. J Biol Chem 277: 31818–31825. doi:10.1074/jbc.M203969200.

33. Sgambato V, Pagès C, Rogard M, Besson M, Caboche J (1998) Extracellular signal-regulated kinase (ERK) controls immediate early gene induction on corticostriatal stimulation. J Neurosci 18: 8814–8825.

34. Falkenburger BH, Jensen JB, Hille B (2010) Kinetics of M1 muscarinic receptor and G protein signaling to phospholipase C in living cells. J Gen Physiol 135: 99–114. doi:10.1085/jgp.200910345.

35. Marcellino D, Kehr J, Agnati LF, Fuxe K (2011) Increased affinity of dopamine for D(2) -like versus D(1) -like receptors. Relevance for volume transmission in interpreting PET findings. Synapse 203: 196–203. doi:10.1002/syn.21501.

36. Chan P, Gabay M, Wright F a, Kan W, Oner SS, et al. (2011) Purification of heterotrimeric G protein alpha subunits by GST-Ric-8 association: primary characterization of purified G alpha(olf). J Biol Chem 286: 2625–2635. doi:10.1074/jbc.M110.178897.

37. Guo Z, Ahmadian MR, Goody RS (2005) Guanine Nucleotide Exchange Factors Operate by a Simple Allosteric Competitive Mechanism. Biochemistry 44: 15423–15429. doi:10.1021/bi0518601.

38. Lenzen C, Cool RH, Prinz H, Wittinghofer A (1998) Kinetic Analysis by Fluorescence of the Interaction between Ras and the Catalytic Domain of the Guanine Nucleotide Exchange Factor Cdc25Mm. Biochemistry 2960: 7420–7430.

39. Traut TW (1994) Physiological concentrations of purines and pyrimidines. Mol Cell Biochem 140: 1–22.

40. Hein P, Rochais F, Hoffmann C, Dorsch S, Nikolaev VO, et al. (2006) Gs activation is time-limiting in initiating receptor-mediated signaling. J Biol Chem 281: 33345–33351. doi:10.1074/jbc.M606713200.

41. Masuho I, Xie K, Martemyanov K a (2013) Macromolecular composition dictates receptor and G protein selectivity of regulator of G protein signaling (RGS) 7 and 9-2 protein complexes in living cells. J Biol Chem 288: 25129–25142. doi:10.1074/jbc.M113.462283.

42. Lavezzari G, Mccallum J, Lee R, Roche KW (2003) Differential binding of the AP-2 adaptor complex and PSD-95 to the C-terminus of the NMDA receptor subunit NR2B regulates surface expression. Neuropharmacology 45: 729–737. doi:10.1016/S0028-3908(03)00308-3.

43. Lavezzari G, Mccallum J, Dewey CM, Roche KW (2004) Subunit-Specific Regulation of NMDA Receptor Endocytosis. J Neurosci 24: 6383–6391. doi:10.1523/JNEUROSCI.1890-04.2004.

44. Prybylowski K, Chang K, Sans N, Kan L, Vicini S, et al. (2005) The synaptic localization of NR2B-containing NMDA receptors is controlled by interactions with PDZ proteins and AP-2. Neuron 47: 845–857. doi:10.1016/j.neuron.2005.08.016.

45. Wang J, Carnicella S, Phamluong K, Jeanblanc J, Ronesi JA, et al. (2007) Ethanol Induces Long-Term Facilitation of NR2B-NMDA Receptor Activity in the Dorsal Striatum: Implications for Alcohol Drinking Behavior. J Neurosci 27: 3593–3602. doi:10.1523/JNEUROSCI.4749-06.2007.

46. Li Y-C, Liu G, Hu J-L, Gao W-J, Huang Y-Q (2010) Dopamine D1 receptor-mediated enhancement of NMDA receptor trafficking requires rapid PKC-dependent synaptic insertion in the prefrontal neurons. J Neurochem 114: 62–73. doi:10.1111/j.1471-4159.2010.06720.x.

47. Spoerner M, Hozsa C, Poetzl J a, Reiss K, Ganser P, et al. (2010) Conformational states of human rat sarcoma (Ras) protein complexed with its natural ligand GTP and their role for effector interaction and GTP hydrolysis. J Biol Chem 285: 39768–39778. doi:10.1074/jbc.M110.145235.
