# Supplementary material for: Segregation and Crosstalk of D1 Receptor-Mediated Activation of ERK in Striatal Medium Spiny Neurons upon Acute Administration of Psychostimulants
Source: PLoS Comput Biol. 2014 Jan 30;10(1):e1003445. doi: 10.1371/journal.pcbi.1003445 (PMC3907292; doi:10.1371/journal.pcbi.1003445)
Supplement: Text S1 — Quantifying protein marker changes in heterogeneous samples. (PDF) [file pcbi.1003445.s008.pdf]

Text S1: Quantifying protein marker changes in heterogeneous samples

The two most common methods to measure changes in the phosphorylation state of a protein marker in a tissue sample are immunohistochemistry and immunoblotting. Immunohistochemistry is commonly used to measure the proportion of positive cells and other aspects related to the special distribution of the modified protein. Immunoblotting is also used for detecting if a change occurred and it allows quantitative estimations. However, in its most common implementation, immunoblotting underestimates the variations in the levels of the modified protein if most cells in the sample contribute to the total pool, but just a fraction of them contributes to the modified marker after the treatment. In order to make a more accurate quantitative estimate of the change in the marker levels in the fraction of responsive cells, immunohistochemistry and immune-blotting can be combined as follows, but first some conventions.

- a^+ and a^- are the **amount** of phosphorylated species in immunohistochemistry positive and negative single cells, respectively. Note that *negative* cells are assumed to have a non-negligible amount of phosphorylated species but below the threshold used.
- N^+ and N^- are the **number** of cells positive and negative for the phosphorylated marker as seen by immunohistochemistry, respectively. N is the total amount of cells in the sample carrying the protein marker so that $N = N^+ + N^-$.
- T, A, IA are the total, the phosphorylated and the non-phosphorylated **amounts** of the species in the sample measured with immunoblotting. T = A + IA
- $f = N^+/N$ is the **fraction** of positive cells in the tissue as measured with immunohistochemistry, relative to the total which have the protein.
- Superscripts + and – stand for positive and negative by immunohistochemistry. Superscripts c and t stand for control and treatment (see below).

The question is then what is the value of,

$$Activation\ level = \frac{a^+}{a^-} \qquad \text{Eq.A1}$$

that is, how many times the level of the phosphorylated species increases when a cell switch from negative to positive by immunohistochemistry. The amount of phosphorylated species is estimated as (Eq.A2),

$$A = g \cdot N \cdot (f \cdot a^+ + (1 - f) \cdot a^-) \qquad \text{Eq.A2}$$

$$T = h \cdot N \qquad \text{Eq.A3}$$

Here g and h are scaling factors from protein concentration to immunoblot intensities for the phosphorylated and the non-phosphorylated species, respectively. Computing the treatment induced change relative to the control after assuming that there is no change in the total amount of the protein due to the treatment ($T^t = T^c$),

$$\frac{\frac{A^t}{T^t}}{\frac{A^c}{T^c}} = \frac{A^t}{A^c} = \frac{f^t \cdot a^+ + (1-f^t) \cdot a^-}{f^c \cdot a^+ + (1-f^c) \cdot a^-} = W \qquad \text{Eq.A4}$$

Rearranging Eq.A4 we get,

$$\frac{a^+}{a^-} = \frac{W\cdot(1-f^c)-(1-f^t)}{(f^t-W\cdot f^c)} \qquad \text{Eq.A5}$$

If $f^c << 1$, this is, there are almost no positive cells as seen by immunohistochemistry in the control sample,

$$\frac{a^+}{a^-} = \frac{W-1+f^t}{(f^t-W\cdot f^c)} \qquad \text{Eq.A6}$$

The following examples illustrate the procedure

Example 1: ERK upon APA in the striatum

- The striatum has several cell types: D1R+MSN, D2R+MSN, several interneurons types, glia etc. MSNs make more than 90% of all neurons and glia could be around a 1/4 to 1/3 of all cells [1]. Thus the MSN D1R+ population is around 1/3.
- ERK is expressed in all of these cell types, possibly at similar levels. This is the total pool.

**Drug**	f^c	f^t	**W**	a^+/a^-	**Reference**
D-Amphetamine 10mg/Kg	0.1	0.3	2.4	28	[2]
Cocaine 20mg/Kg	0.1	0.3	2.1	15	[2,3]
Cocaine 20mg/Kg	0.099	0.033	2.1	40	[3,4]

Example 2: STEP upon APA in the striatum

- Contrary to ERK, STEP is not present in striatal glia but just in medium spiny neurons [5]. Thus the fraction of D1R+MSNs in the STEP-expressing cell population is 0.5.
- W = 2.6 for STEPp upon APA (D-amphetamine, 10 mg/Kg) [2].
- There is no information about the number of STEPp positive neurons in the control and in the treatment. All D1R+MSNs are ERKpp positive upon APA [2], and assuming that this subpopulation is STEPp positive, ft = 0.5, and that in basal conditions the fraction of STEPp positive MSNs is negligible, fc ~ 0, then $a^+/a^- = 4.2$, and the basal levels of STEPp in MSNs is around 0.2 of the total (Figure 6a in [2]).

Example 3: D32p34 upon treatment of striatal slices with D1R agonists.

- Like STEP, striatal D32 is present just in MSNs [4].
- W = 6 upon treatment of striatal slices with D1R [6].
- The basal level of D32p34 is rather low, thus $f^c \sim 0$, and assuming that all D1R+MSNs become positive for D32p34 upon treatment of striatal slices with D1R agonists ($f^t = 0.5$), $a^+/a^- = 11$.

Example 4: GluR1p845 upon treatment with cocaine (20mg/Kg)

- GluR1 is mainly a neuronal protein.
- W = 6 upon APA (cocaine 20mg/Kg) [7].
- The basal level of GluR1p845 is very low, thus $f^c \sim 0$, and assuming that all D1R+MSNs become positive for GluR1p845 upon APA ($f^t = 0.5$), $a^+/a^- = 11$.

Limitations

Despite this approach constitute an improvement over the direct use of immunoblot estimates, it still have some limitations. While the resulting a^+/a^- are near double or more the immunoblot estimate this could still be an underestimation specially in those cases where we have assumed that the target population is completely positive and the fraction of positive cells in basal conditions is nearly 0. More importantly, the two-state assumption where a neuron in the basal and positive states have fixed levels of modified markers, a^- and a^+ respectively, is clearly a simplification. While two state activation is considered to be widespread either in the form of bistable systems or just non-hysteresis ultrasensitive systems (i.e. zero-order ultrasensitivity), the transition from one state to the other is not instantaneous and at any point during the course of the activation the level of modified marker in a cell would be somewhere between a^- and a^+. On the other hand the a- and a+ may not be the same for every target cell in the sample [8]. Furthermore, even for the same system the time course of modification followed by immunohistochemistry in terms of fraction of positive cells is not tightly correlated to the immunoblot measurement [3,4].

According to Eq.A5 and Eq.A6, the ratio a^+/a^- has a vertical asymptote at $f^c = f^t/W$. This means that under the assumptions of this derivation there cannot be an increase in the intensity of immunoblotting bands after an activating treatment if there is not an increase in the fraction of positive cells. As the fraction of positive cells before and after the treatment becomes closer, the change in the amount of active marker from a^- to a^+ growth faster to back up a given W. These limitations arise from the two-states and instantaneous switch assumptions.

Finally, we have considered just cell types with the soma in the striatum, but there are extensive axonal projections from dopaminergic and cholinergic neurons which could constitute sources of inaccuracy. This would need further analysis.

References

1. Matamales M, Bertran-Gonzalez J, Salomon L, Degos B, Deniau J-M, et al. (2009) Striatal medium-sized spiny neurons: identification by nuclear staining and study of neuronal subpopulations in BAC transgenic mice. PLoS One 4: e4770.

2. Valjent E, Pascoli V, Svenningsson P, Paul S, Enslen H, et al. (2005) Regulation of a protein phosphatase cascade allows convergent dopamine and glutamate signals to activate ERK in the striatum. PNAS 102: 491–496.

3. Valjent E, Bertran-Gonzalez J, Aubier B, Greengard P, Hervé D, et al. (2010) Mechanisms of locomotor sensitization to drugs of abuse in a two-injection protocol. Neuropsychopharmacology 35: 401–415.

4. Bertran-Gonzalez J, Bosch C, Maroteaux M, Matamales M, Hervé D, et al. (2008) Opposing patterns of signaling activation in dopamine D1 and D2 receptor-expressing striatal neurons in response to cocaine and haloperidol. J Neurosci 28: 5671–5685.

5. Lombroso PJ, Naegele J, Sharma E, Lerner M (1993) A protein tyrosine phosphatase expressed within dopaminoceptive neurons of the basal ganglia and related structures. J Neurosci 13: 3064–3074.

6. Nishi A, Snyder GL, Greengard P (1997) Bidirectional regulation of DARPP-32 phosphorylation by dopamine. J Neurosci 17: 8147–8155.

7. Snyder GL, Allen PB, Fienberg AA, Valle CG, Huganir RL, et al. (2000) Regulation of Phosphorylation of the GluR1 AMPA Receptor in the Neostriatum by Dopamine and Psychostimulants In Vivo. J Neurosci 20: 4480–4488.

8. Cohen-Saidon C, Cohen AA, Sigal A, Liron Y, Alon U (2009) Dynamics and Variability of ERK2 Response to EGF in Individual Living Cells. Mol Cell 36: 885–893.
